# When Does Machine Learning Add Value over Theory? Predicting API Solubility in Binary Mixtures with COSMO-RS and DOOIT2 Across Diverse and Homogeneous Systems

**DOI:** 10.3390/molecules31101566

**Published:** 2026-05-08

**Authors:** Maciej Przybyłek, Tomasz Jeliński, Adrian Drużyński, Piotr Cysewski

**Affiliations:** 1 Department of Physical Chemistry, Faculty of Pharmacy, Collegium Medicum of Bydgoszcz, Nicolaus Copernicus University in Toruń, Kurpińskiego 5, 85-950 Bydgoszcz, Poland; tomasz.jelinski@cm.umk.pl (T.J.); 288428@stud.umk.pl (A.D.); 2 Institute of Advanced Studies, Nicolaus Copernicus University in Toruń, Wileńska 4, 87-100 Toruń, Poland

**Keywords:** solubility, binary mixtures, machine learning, COSMO-RS, 4-formylmorpholine, API, phenolic acids, QSPR, DOOIT2, applicability domain

## Abstract

Predicting the solubility of active pharmaceutical ingredients (APIs) in binary aqueous-organic mixtures is critical for formulation design, yet remains challenging. Physics-based models such as COSMO-RS provide a solid theoretical foundation but often struggle with non-ideal mixing behavior in complex systems. This study asks a practical question: when does machine learning actually add value beyond established theory? We compared COSMO-RS with DOOIT2 (Dual-Objective Optimization with Iterative Feature Pruning), a hybrid COSMO-RS/machine-learning correction workflow, across two complementary datasets: 85 structurally diverse APIs and related formulation-relevant compounds (10,140 data points) and 37 acid-centered solutes (6030 data points). The datasets also incorporate newly measured solubilities of lidocaine, benzocaine, and vanillic acid in aqueous 4-formylmorpholine mixtures. DOOIT2 employs rigorous API-out Structured Group K-Fold validation with fold-specific ensemble models to ensure realistic assessment of generalization to unseen compounds. The obtained results are dataset-dependent. For the homogeneous acid series, COSMO-RS already delivers strong predictive performance (*RMSD* = 0.321, *R*^2^ = 0.925), and DOOIT2 brings no meaningful improvement (*RMSD* = 0.310, *R*^2^ = 0.923). In contrast, for the diverse API set, DOOIT2 reduces *RMSD* from 0.686 to 0.527 and increases *R*^2^ from 0.829 to 0.849. Residual analysis indicates that prediction uncertainty is driven primarily by the low-solubility region rather than by a simple monotonic dependence on molecular weight alone. These findings delineate the practical boundaries of machine-learning assistance in solubility prediction and offer clear guidance for formulation scientists.

## 1. Introduction

The solubility of active pharmaceutical ingredients (APIs) in binary aqueous-organic solvent mixtures is one of the most practically important physicochemical properties in drug development. It directly governs key processes such as formulation design, selection of crystallization protocols, dissolution behavior in the gastrointestinal tract, and ultimately the bioavailability of new drug candidates. In many cases, the choice of co-solvent system can dramatically enhance the apparent solubility and dissolution rate of poorly water-soluble compounds, thereby improving their therapeutic potential while reducing the risk of formulation-related failures later in development [1,2,3,4,5].

It is therefore no surprise that considerable research efforts are devoted to improving and predicting API solubility in such systems [6,7,8]. However, reliable solubility data across the full composition range are essential not only for optimizing final dosage forms but also for supporting rational solvent selection during early formulation screening and process scale-up [9,10,11].

In the earliest stages of discovery, when hundreds or even thousands of candidate compounds must be evaluated, exhaustive experimental screening across the full composition and temperature range is simply not feasible. As a result, reliable predictive models are no longer a luxury but a practical necessity. They enable rapid prioritization of promising candidates, guide solvent selection, and significantly reduce the enormous material and time costs associated with purely empirical testing [12,13,14,15].

For many years, physics-based approaches, particularly the Conductor-like Screening Model for Real Solvents (COSMO-RS), have been widely used for such predictions [16,17,18]. In pharmaceutical solubility research, COSMO-RS remains an important reference framework because it provides a theoretically grounded route to solubility estimation and is applicable even when experimental information is limited [16,19,20,21]. For this reason, it offers a natural baseline against which the added value of data-driven correction can be evaluated.

However, despite its theoretical rigor, COSMO-RS does not perform uniformly across all systems, particularly in the context of mixed-solvent solubility prediction. Evidence from previous studies demonstrates that, while the model can provide accurate results for certain systems, it may exhibit substantial deviations in others, especially when complex or non-ideal interactions dominate. For example, in the case of ethenzamide, accurate prediction required extension of the COSMO-RS framework through explicit inclusion of intermolecular interactions (COSMO-RS-DARE), leading to a substantial improvement in predictive performance (*R*^2^ = 0.886, MAE = 0.034 in log units) [22]. In contrast, for structurally diverse APIs and more complex solvent environments, baseline COSMO-RS predictions were found to be significantly less accurate, with reported performance as low as *R*^2^ = 0.327 and MAE = 0.778 [23]. Intermediate cases, such as edaravone (*R*^2^ = 0.760, MAE = 0.310) and theophylline (*R*^2^ = 0.449, MAE = 0.048), further illustrate that predictive reliability strongly depends on both solute structure and solvent environment [23]. All reported metrics refer to logarithmic mole fraction solubility. Similar observations have been reported across related systems studied within the COSMO-RS framework. These findings indicate that COSMO-RS is not inherently unreliable, but rather that its performance is system-dependent, with limitations becoming apparent in cases involving complex intermolecular interactions or heterogeneous chemical spaces. The research gap addressed in the present study is therefore not whether COSMO-RS is generally useful, but under what conditions it remains sufficient on its own and when additional correction strategies, such as hybrid data-driven approaches, become justified.

In response to these limitations, machine-learning methods have gained increasing attention in recent years. By learning empirical patterns directly from experimental data, ML models can, in principle, identify and correct for systematic deficiencies in purely theoretical descriptions [24,25,26]. Rather than replacing physics-based models, modern approaches often adopt a hybrid strategy, i.e., using COSMO-RS-derived descriptors as physically meaningful inputs while allowing data-driven components to refine predictions where theory alone falls short [27,28,29]. In this context, DOOIT2 (Dual-Objective Optimization with Iterative Feature Pruning) should be understood as a hybrid COSMO-RS/machine-learning correction workflow rather than an independent predictive model. COSMO-RS predictions (RefSol) are retained as the physically grounded baseline, while machine learning is used to capture systematic residual patterns associated with complex solvation behavior that are not fully described by the theoretical model. This broader conceptual framework is also supported by recent published studies specifically focused on solubility prediction, including the combination of molecular thermodynamics with machine learning for drug solubility in solvents, hybrid semi-mechanistic regression for crystallization-oriented solubility modeling, and comparative thermodynamic/hybrid modeling in pharmaceutical solubility systems [30,31,32].

Over the past decade, a variety of machine-learning architectures, from random forests and gradient boosting to deep neural networks and graph convolutional networks, have been applied to aqueous and mixed-solvent solubility prediction [33,34,35]. While many of these studies report impressive statistics on random train-test splits, a fundamental question has remained largely unanswered: under what conditions does machine learning genuinely improve upon well-established theory, and when does it merely reproduce or even degrade theoretical performance?

Answering this question requires more than another benchmark on a single dataset. It demands systematic comparison across chemically distinct solute classes, consistent and rigorous validation protocols, and explicit evaluation of generalization to truly novel compounds, i.e., the most relevant scenario to pharmaceutical research. In the present work we address exactly this challenge by comparing COSMO-RS with the newly developed DOOIT2 workflow on two deliberately contrasting datasets.

The first dataset (API) contains 85 structurally diverse active pharmaceutical ingredients and related bioactive or formulation-relevant compounds spanning multiple therapeutic classes and chemical scaffolds. It yielded 10,140 solubility records and primarily covers aqueous mixtures with polar aprotic and related organic cosolvents. The second dataset (PhAAc) comprises 37 chemically narrower acid-centered solutes, including phenolic acids, substituted benzoic and cinnamic acids, and related carboxylic or acidic drug-like compounds. This dataset yielded 6030 solubility records and provides broader solvent-space coverage, including aqueous and non-aqueous binary mixtures with alcohols, esters, ketones, nitriles, glycols, carboxylic acids, ethers, and related solvent classes. The two datasets therefore provide complementary test cases: a chemically diverse API-centered set and an acid-centered set with broader solvent coverage. Importantly, the present study incorporates new experimental solubility measurements for lidocaine, benzocaine, and vanillic acid in aqueous mixtures of 4-formylmorpholine, a relatively underexplored but chemically informative solvent system that combines features of dipolar aprotic proton-acceptor media with pronounced preferential-solvation behavior in aqueous mixtures. These new data expand the available solubility literature and provide an additional test case for model transferability to a solvent system that was not heavily represented during training.

To ensure fair and realistic assessment of generalization, we introduced DOOIT2, an evolution of our previously published DOOIT methodology [23]. The key innovation lies in the adoption of API-out Structured Group K-Fold (SGKF) cross-validation combined with fold-specific ensemble models. Unlike conventional random splits, which can inadvertently place structurally similar compounds in both training and test sets, API-out validation guarantees that every prediction for a given compound is made by a model that has never seen any data for that compound. This approach mirrors the real-world situation faced by formulation scientists who must predict the behavior of entirely new molecules. By training a separate ensemble for each validation fold, DOOIT2 also provides a more honest estimate of uncertainty and model stability.

In this study we therefore pose three concrete questions. First, does DOOIT2 outperform COSMO-RS, and if so, for which type of solute class? Second, what are the clear boundaries of the model’s applicability domain, that is which compounds are reliably predicted and which fall outside its learned relationships? Third, how does the methodological shift from random splits to API-out validation affect both performance metrics and the confidence we can place in the results?

By systematically addressing these questions, the present work offers (i) new experimental solubility data for aqueous 4-formylmorpholine systems, involving a less-explored dipolar aprotic solvent with relevance to greener solvent-selection and solvent-replacement strategies; (ii) a systematic comparison of COSMO-RS and ML across chemically distinct datasets; (iii) a rigorous methodology (DOOIT2) for developing and validating generalizable quantitative structure-property relationship (QSPR) models; (iv) a clear delineation of the model’s applicability domain, identifying compounds where predictions are reliable and those where they are not; and (v) a practical tool for formulation scientists seeking to reduce experimental screening in early-stage development. Such insights are essential if we are to move beyond the current “ML versus theory” debate toward a more rational, mechanism-informed strategy for solubility prediction in pharmaceutical science.

## 2. Results and Discussion

### 2.1. Experimental Solubility

**Figure 1:**
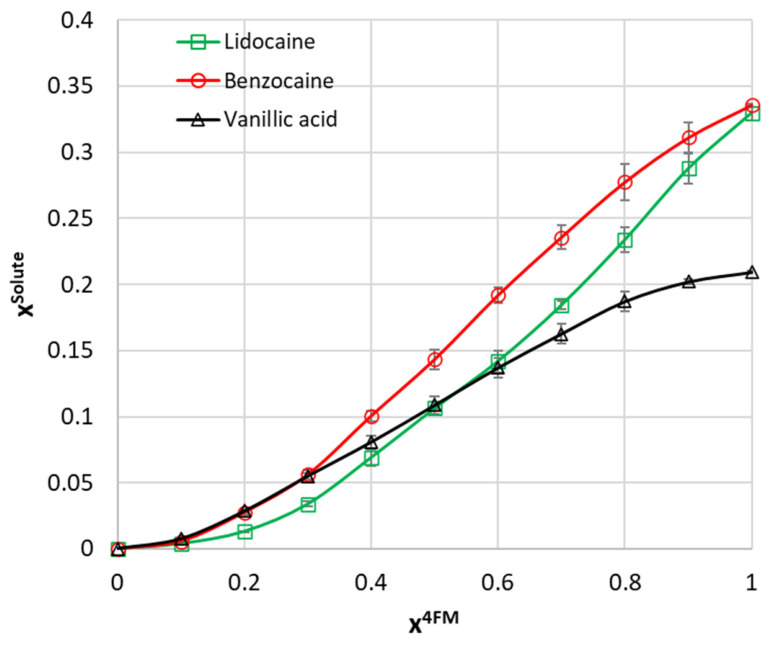
Experimental mole fraction solubility (x^solute^) of lidocaine, benzocaine, and vanillic acid in binary 4-formylmorpholine (4FM)-water mixtures at 298.15 K, plotted as a function of the mole fraction of 4FM in the solute-free solvent mixture (x^4FM^). Error bars denote standard deviations. The complete numerical solubility data are provided in the Supplementary Materials.

**Figure 2:**
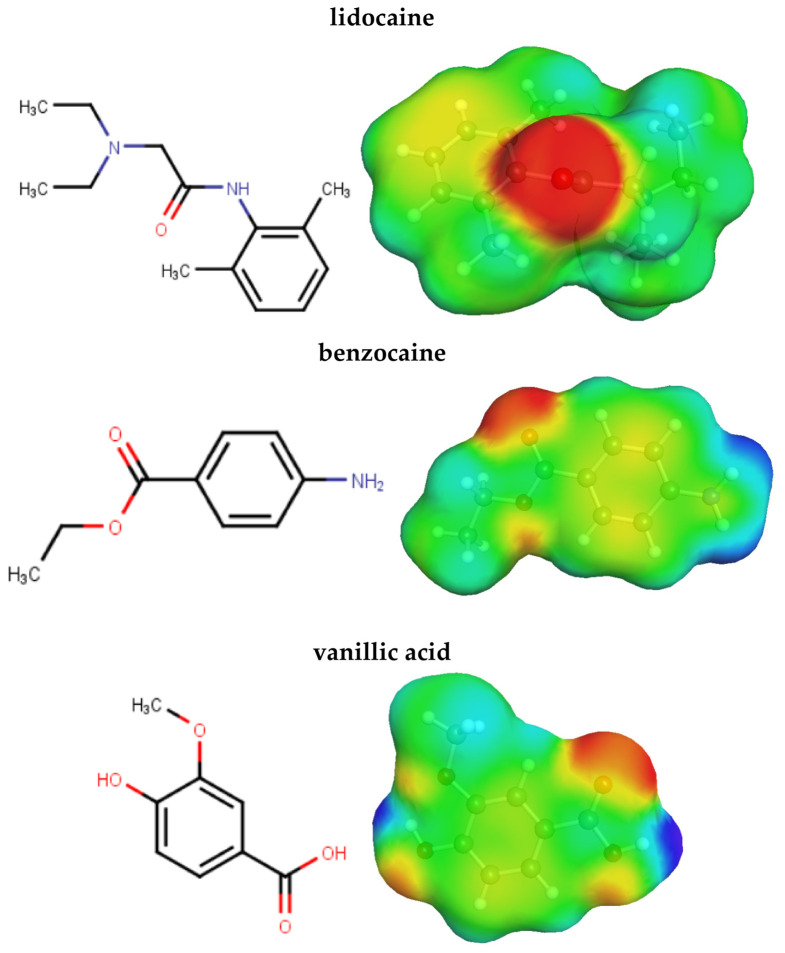
Structural representation of lidocaine, benzocaine, and vanillic acid in the form of a 2D sketch and charge density distribution.

The experimental solubility of lidocaine, benzocaine, and vanillic acid in 4-formylmorpholine (4FM)-water mixtures at 298.15 K is presented in Figure 1. Full numerical solubility data are provided in the Supplementary Materials in Table S1.1. These three compounds were selected because they represent distinct combinations of functional groups relevant to solvation in mixed solvents. The differences in polarity and intermolecular interaction potential are further illustrated by COSMO-RS σ-surfaces shown in Figure 2, which highlight the distinct distribution of charge density regions for each compound. Although lidocaine and benzocaine are both aromatic local anesthetics, they are not structurally equivalent. Lidocaine contains both amide and tertiary amine groups and has a more flexible structure, whereas benzocaine is a simpler aromatic ester with lower polarity. Vanillic acid differs more markedly because it contains carboxyl, hydroxyl, and methoxy groups, which together provide a distinct balance of polarity and intermolecular interactions.

The choice of 4FM also warrants a brief comment. Compared with routinely used dipolar aprotic solvents such as DMSO, DMF, and acetonitrile, 4FM remains less explored in pharmaceutical solubility studies. The greener-solvent aspect of this medium is relevant and has been noted in earlier solvent-selection and synthesis-oriented studies [36,37,38,39,40,41]. However, this was not the sole reason for its selection in the present work. From a physicochemical perspective, 4FM is a high-boiling dipolar aprotic proton-acceptor solvent, and its aqueous mixtures have been shown to display pronounced composition-dependent solvatochromic and preferential-solvation behavior [42]. This made it a useful test medium for the present study, because it extends the solubility mapping of structurally distinct solutes to an underrepresented class of aqueous proton-acceptor mixed solvents.

As shown in Figure 1, the mole fraction solubility increased steadily with increasing x_4FM_ for all three compounds, and no local maximum was observed within the investigated composition range. In pure water, lidocaine and vanillic acid showed comparable solubility, with x_solute_ values of 3.13 × 10^−4^ and 2.91 × 10^−4^, respectively, whereas benzocaine was the least soluble compound, with x_solute_ = 1.06 × 10^−4^. In pure 4FM, benzocaine showed the highest solubility, with x_solute_ = 3.36 × 10^−1^, followed closely by lidocaine, with x_solute_ = 3.30 × 10^−1^, while vanillic acid remained distinctly less soluble, with x_solute_ = 2.09 × 10^−1^.

### 2.2. Dataset Characteristics

To systematically evaluate the predictive capabilities of COSMO-RS and the DOOIT2 machine learning framework, two complementary datasets were assembled, each designed to probe different dimensions of generalization performance. The datasets differ fundamentally in their chemical composition, solvent coverage, and the nature of the generalization challenge they represent. The first dataset, hereafter referred to as API, comprises 85 structurally diverse solutes centered on active pharmaceutical ingredients and related bioactive or formulation-relevant compounds. The set spans multiple chemical classes, including anti-infective agents, sulfonamides and related antimicrobial compounds, xanthines and other polar heterocycles, aromatic amides and analgesic-type compounds, local anesthetics, nitro- and halo-substituted aromatics, triazoles and other N-heterocycles, and selected sugars or polyol-related compounds. This chemical diversity was intentionally selected to challenge the generalization capability of predictive models because the dataset contains compounds with limited structural similarity to one another. Solubility measurements in the API dataset yielded 10,140 records collected for aqueous mixtures with polar aprotic and related organic cosolvents. The solvent space represented in the curated workbook includes 4-formylmorpholine, dimethylformamide, dimethyl sulfoxide, N-methyl-2-pyrrolidone, acetonitrile, 1,4-dioxane, acetone, formamide, N-methylformamide, and tetrahydrofuran in combination with water. Notably, the compiled datasets include new experimental measurements for lidocaine, benzocaine, and vanillic acid in 4-formylmorpholine-water mixtures, reported here for the first time. These data expand the limited literature on 4FM as a pharmaceutical co-solvent and provide an isothermal test case for model performance in a relatively unexplored solvent system, rather than a full thermodynamic characterization of this solvent pair. Besides equilibrium solubility itself, possible solid-state transformations during equilibration may also influence the interpretation of compound-specific behavior. This aspect, however, was beyond the main scope of the present comparative modeling study. The second dataset, referred to as PhAAc, comprises 37 acidic solutes and is chemically narrower than the API set, although it is not limited to a single benzoic- or cinnamic-acid scaffold. In addition to phenolic acids and substituted benzoic or cinnamic acids, it also contains heteroaromatic and dicarboxylic acids, amino- and hydroxy-acids, and selected acidic drug-like compounds. This composition still provides a substantially more constrained solute space than the API dataset and therefore remains suitable for evaluating model behavior within a chemically narrower domain. Solubility measurements in the PhAAc dataset yielded 6030 records collected across a broader set of aqueous and non-aqueous binary solvent systems. The solvent space extends well beyond DMF, DMSO, and 4FM and includes alcohols, esters, ketones, nitriles, glycols, carboxylic acids, ethers, and related mixed-solvent systems. Within the acid-centered PhAAc dataset, the newly measured vanillic-acid system provides the corresponding experimentally characterized 4FM-containing case. Taken together, the API and PhAAc datasets provide complementary testbeds for evaluating the performance of COSMO-RS and DOOIT2 under distinct generalization scenarios.

### 2.3. COSMO-RS Baseline Performance

Before evaluating the machine learning models, we established a baseline using COSMO-RS predictions. All calculations were performed using the reference solvent approach, wherein experimental solubility values in the neat solvents, specifically both neat components of the binary solvent system, were supplied as input. This approach effectively anchors the predictions to experimental endpoints and isolates the ability of the model to capture non-ideal mixing behavior across the composition range. Moreover, this setup circumvents the need to provide fusion data, including melting temperature, enthalpy of fusion, and heat capacity differences, which are often unavailable, inconsistent, or problematic for many compounds of pharmaceutical interest.

The use of fusion data in traditional solubility modeling presents several well-documented challenges. First, reliable measurements of melting properties require highly pure crystalline samples and specialized techniques such as differential scanning calorimetry, which may not be available for all compounds, particularly during early-stage drug discovery when only milligram quantities are accessible. Second, many compounds exhibit polymorphism, defined as the existence of multiple crystalline forms with distinct thermodynamic properties, and the fusion data obtained may correspond to a metastable polymorph rather than the thermodynamically stable form relevant to solubility measurements. This ambiguity introduces systematic errors when fusion data are used to convert activity coefficients to absolute solubility. Third, for compounds that decompose before melting or exhibit glass transitions rather than true melting, conventional fusion data are either unattainable or physically meaningless. Fourth, literature compilations of thermophysical data, such as those by Acree and Chickos [43,44], have demonstrated that reported fusion properties may exhibit substantial variability across independent studies, particularly for compounds prone to polymorphism or thermal decomposition. Reported melting temperatures can differ by several degrees, and enthalpy values may vary significantly depending on experimental conditions and sample history. Such inconsistencies introduce additional uncertainty when fusion data are used to derive solubility from thermodynamic relationships. This variability is particularly problematic in pharmaceutical systems, where polymorphism and metastable forms are common. By adopting the reference solvent approach, we bypass these complications entirely and anchor predictions directly to experimentally measured solubility in neat solvents at the temperature of interest. This focuses the modeling effort on the critical challenge of capturing non-ideal mixing behavior across the composition range, independent of the uncertainties inherent in fusion property estimation.

**Figure 3:**
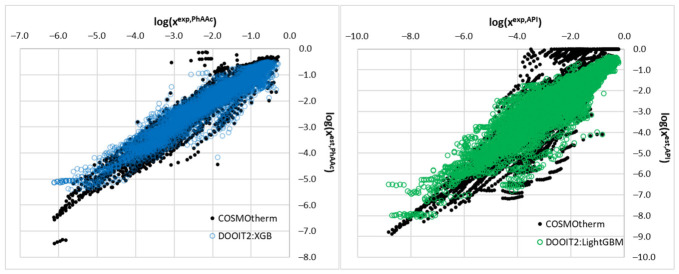
Parity plots of experimental versus COSMO-RS predicted solubility (log mole fraction) for (**left**) the PhAAc dataset (37 phenolic and carboxylic acids, 6030 data points) and (**right**) the API dataset (85 diverse APIs, 10,140 data points). The solid line represents ideal agreement (y = x). For the PhAAc dataset, COSMO-RS achieves *RMSD* = 0.321 and *R*^2^ = 0.925, indicating good overall predictive performance. For the API dataset, performance degrades markedly (*RMSD* = 0.686, *R*^2^ = 0.829), with substantial scatter around the ideal line, reflecting the greater challenge posed by structurally diverse solutes.

As shown in Figure 3 (left panel), COSMO-RS demonstrated strong predictive performance for the 37 phenolic and carboxylic acids. The root mean square deviation (*RMSD*) across all 6030 data points was 0.321 log units. The coefficient of determination (*R*^2^ = 0.925) indicates that 92.5% of the variance in experimental log solubility is explained by the COSMO-RS predictions. The combination of moderate prediction error and high *R*^2^ confirms that, for this solute class, the reference-solvent approach captures the overall solvation trends across the full composition range well. The relatively narrow spread of solubility values in this dataset should also be kept in mind when interpreting the correlation coefficient.

In the case of the API dataset, COSMO-RS exhibited substantially weaker performance, as illustrated in Figure 3 (right panel). The *RMSD* increased to 0.686 log units, reflecting the greater challenge posed by structurally diverse solutes. At the same time, the *R*^2^ value decreased to 0.829, and the scatter around the ideal line was considerably larger, with several compounds exhibiting pronounced systematic deviations. This finding is significant because, despite anchoring calculations to experimental neat-solvent solubility endpoints, the physics-based model does not fully capture the complex and non-additive interactions governing solubility in binary mixtures for structurally diverse solutes. This establishes a clear rationale for applying machine learning to the more challenging API dataset.

### 2.4. Machine Learning DOOIT2 Models Performance

The contrasting results across the two datasets address the central question of this study by showing that the added value of machine learning is dataset-dependent. However, the determining factor is not simply dataset diversity or homogeneity as abstract categories, but rather the underlying chemical scope of the solutes and the complexity of their solvation behavior. The PhAAc dataset represents a chemically narrower acid-centered domain, dominated by phenolic, benzoic, cinnamic, and related carboxylic or acidic compounds with more recurrent interaction patterns. This chemical coherence helps explain why COSMO-RS already captures the main solvation trends with good accuracy. In contrast, the API dataset encompasses a broader range of chemical scaffolds, from relatively simple aromatic amides to heterocyclic and more structurally diverse drug-like molecules. These chemically varied structures present distinct solvation challenges that are not captured uniformly by a single theoretical baseline.

#### 2.4.1. Model Selection and Validation

The DOOIT2 framework was applied to both datasets using API-out Structured Group K-Fold (SGKF) cross-validation to ensure a rigorous assessment of generalization to unseen compounds. This approach represents a substantial methodological advancement over our previous DOOIT implementation [23,45], which relied on repeated random 80/20 splits. Although random split validation is appropriate for assessing interpolative performance within a chemical series, it systematically overestimates generalization to novel compounds because structurally similar compounds may appear in both the training and test sets. By enforcing chemical separation at the solute level and ensuring that all measurements for a given API are assigned exclusively to either the training or the validation fold, DOOIT2 provides a more honest and realistic assessment of predictive capability. The choice of regression algorithm was not fixed a priori. Instead, multiple ensemble methods were benchmarked for each dataset, and the final selection was based on cross-validated performance under the API-out SGKF framework. As a result, LightGBM (version 4.6.0) was selected for the API dataset, whereas XGBoost (version 3.2.0) performed best for the PhAAc dataset.

For the API dataset, LightGBM with the set 3 feature configuration emerged as the optimal model, whereas XGBoost performed best for the PhAAc dataset.

#### 2.4.2. Descriptor Selection for DOOIT2 Models

The hybrid strategy adopted in this work does not alter the COSMO-RS formalism. The COSMO-RS prediction (RefSol) is retained as the baseline, while additional descriptors derived from COSMO-RS outputs are used exclusively as inputs to a separate empirical correction model. These descriptors should therefore be interpreted as features in a machine learning context rather than as modifications of the underlying thermodynamic equations. An analysis of the features selected by the fold-specific XGBoost models for the PhAAc dataset reveals patterns in both consistency and variability across the folds (Table S4.1 in the Supplementary Materials). RefSol, which represents the COSMO-RS-predicted solubility, was retained as the baseline predictor, whereas the remaining COSMO-RS-derived energetic and σ-profile descriptors were used only as physically informed inputs in a separate empirical correction layer. The fact that RefSol was selected in all five folds confirms that the machine learning model was built around the baseline COSMO-RS estimate rather than as a replacement for it. The recurrent selection of descriptors such as d_HH2 should therefore be interpreted as an empirical feature-selection trend within the hybrid model, not as a reformulation of the underlying COSMO-RS formalism. Several other descriptors showed a high selection frequency across the folds. The hydrophobic region descriptor d_HH3 and the hydrogen bond acceptor descriptor d_HBA4 were both retained with an 80% frequency. Additionally, the solute van der Waals energy E1_vdW_sat and the solvent hydrogen bonding energy E_HB_solvent were frequently retained with a 60% frequency. This pattern suggests that successful prediction requires integrating information from three complementary domains. These include a baseline theoretical prediction, solute-specific energetic terms capturing van der Waals and hydrogen bonding contributions, and an explicit characterization of the interaction landscape of the solvent mixture through σ-profile-derived descriptors. Notably, the number of selected features varied substantially across the folds, ranging from 4 to 19 descriptors, with corresponding fold MAE values ranging from 0.187 to 0.249. This variability does not indicate model instability but rather reflects the fold-specific ensemble approach. Each fold model is optimized for the particular set of compounds held out for validation, and different chemical subspaces may require different descriptor combinations to achieve an optimal prediction. The fold with the lowest MAE of 0.187, designated as Fold 4, selected a parsimonious set of 7 features. Conversely, the fold with the highest MAE of 0.249, designated as Fold 1, selected 19 features. This suggests that certain chemical subspaces inherently require more complex descriptor combinations to achieve comparable accuracy.

An analysis of the features selected by the fold-specific LightGBM models for the API dataset reveals both striking similarities and notable differences compared to the PhAAc models (Table S4.2 in the Supplementary Materials). As observed with the PhAAc dataset, the baseline descriptor RefSol, representing the COSMO-RS predicted solubility, was selected in all five folds, thereby confirming its foundational role regardless of the solute class. Several descriptors demonstrated an equally high selection frequency. The solute van der Waals energy E1_vdW_sat as well as the σ-potential-derived descriptors for the hydrogen bond donor region d_HBD1 and the hydrophobic region d_HH1 were each selected in all five folds. This consistent selection across all chemical subspaces indicates that these descriptors capture fundamental physicochemical properties essential for predicting solubility in binary mixtures irrespective of the specific APIs held out in each fold. The differential descriptors dE_HB_sat and dE_vdW_sat, which represent the relative difference between solute and solvent hydrogen bonding and van der Waals energies, respectively, were selected in 4 out of 5 folds, resulting in an 80% frequency. Their near-universal presence suggests that the model learns to weight the balance between solute and solvent interaction strengths, which effectively captures competitive solvation effects. The API-specific σ-potential descriptors, namely API_HBA4, API_HBA2, and API_HH2, showed lower selection frequencies of 60% and below. This indicates that while certain regions of the solute charge density profile are important for some chemical subspaces, they are not universally required across all folds. Notably, the number of selected features across the folds ranged from 6 to 10 descriptors, with corresponding fold MAE values ranging from 0.332 to 0.403. In contrast to the PhAAc dataset, where the fold with the highest MAE of 0.249 selected the largest number of features at 19, the API dataset showed no clear correlation between model complexity and fold error. This suggests that for structurally diverse APIs, the optimal descriptor set size is more constrained, and the prediction difficulty may arise from factors beyond a simple descriptor count.

A comparative analysis of the descriptors used in the PhAAc and API models is provided in Table 1. The table summarizes the selection frequency, defined as the percentage of SGKF folds in which each descriptor category was retained, for the XGBoost model trained on the PhAAc dataset and the LightGBM model trained on the API dataset. Descriptor categories group related features based on their physicochemical interpretation. The contrasting patterns reveal that the API model places greater emphasis on solute-solvent differential terms and hydrogen bond donor descriptors, whereas the PhAAc model relies more heavily on solvent mixture properties. Descriptor definitions are provided in Supplementary Tables S3.1 and S3.2, whereas per-fold feature-selection results are provided in Supplementary Tables S4.1 and S4.2.

The most striking difference lies in the differential descriptors dE_HB_sat and dE_vdW_sat, which capture the balance between solute and solvent interaction strengths. These features were selected in 80% of the folds for the API model but in only 20 to 40% of the folds for the PhAAc model. This finding is physicochemically informative because, for structurally diverse APIs, the model appears to use these descriptors to capture how the relative strength of solute–solvent interactions varies across compounds with different functional groups. For the chemically narrower PhAAc dataset, where solutes share a more coherent acidic domain, this relative balance is more predictable and can be captured by simpler descriptor combinations. Similarly, the consistent selection of the hydrogen-bond donor descriptor d_HBD1 in all five API folds, compared with only a 40% frequency in the PhAAc model, indicates that donor-capacity variation contributes more strongly to the API model. This observation is consistent with the chemical composition of the two datasets: the PhAAc dataset is dominated by acidic compounds with more recurrent donor/acceptor patterns, whereas the API dataset spans a wider range of donor capacities.

The descriptor selection patterns for the API model reveal a learning strategy that complements the PhAAc findings. Both models invariably retain the COSMO-RS baseline as a foundation. However, the API model places greater emphasis on solute-solvent differential terms that capture the competitive balance between interactions. This balance constitutes a critical factor for diverse chemical structures where the matching between the API and the solvent varies widely. Furthermore, the API model emphasizes hydrogen bond donor descriptors that reflect the broad range of donor capacities across the API set, alongside the solute van der Waals energy. The consistent selection of this van der Waals energy descriptor across all folds indicates that dispersion interactions are universally important regardless of the specific API. The lower frequency of solvent-specific descriptors in the API model compared to the PhAAc model suggests that when solute diversity is high, the model prioritizes capturing the variability in solute properties over detailed solvent characterization. This prioritization likely occurs because solvent properties are already encoded in the RefSol baseline and the differential terms.

#### 2.4.3. Accuracy of DOOIT2 Models

**Figure 4:**
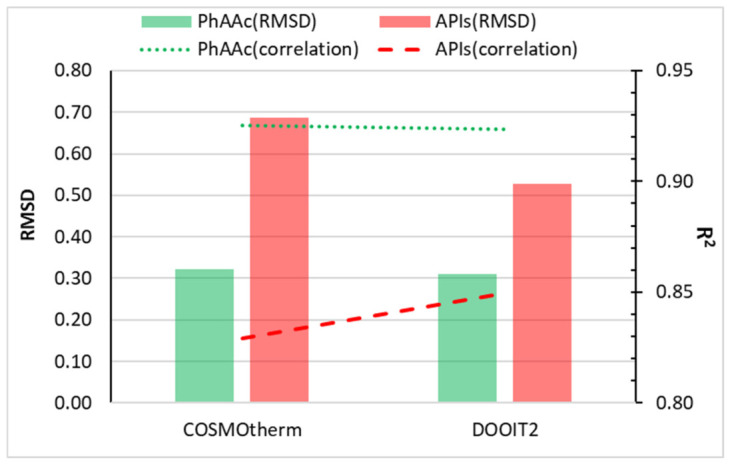
Predictive performance of COSMO-RS and DOOIT2 for the PhAAc and API datasets. Bar height represents *RMSD* (left axis), and line markers indicate *R*^2^ (right axis) for solubility prediction (log mole fraction) in binary aqueous-organic mixtures. For the PhAAc dataset, COSMO-RS and DOOIT2 yielded *RMSD* values of 0.321 and 0.310 and *R*^2^ values of 0.925 and 0.923, respectively. For the API dataset, the corresponding values were 0.686 and 0.527 for *RMSD* and 0.829 and 0.849 for *R*^2^.

The predictive performance of COSMO-RS and DOOIT2 is summarized in Figure 4, which presents a side-by-side comparison of COSMO-RS and DOOIT2 performance. For the PhAAc dataset, DOOIT2 does not provide a meaningful improvement over COSMO-RS, because the *RMSD* changes only slightly from 0.321 to 0.310, while *R*^2^ remains essentially unchanged at 0.925 versus 0.923. This result is consistent with expectation: when solutes share a chemically narrower and more coherent interaction pattern, the quantum-chemical interactions are already captured well by COSMO-RS, leaving little room for additional data-driven refinement. In contrast, for the structurally diverse API dataset, DOOIT2 improves performance relative to COSMO-RS, reducing the *RMSD* from 0.686 to 0.527 and increasing *R*^2^ from 0.829 to 0.849. This improvement remains moderate in absolute terms and should not be overstated. However, it is worth emphasizing that this result was obtained under a rigorous one-API-out validation framework in which the model was tested on compounds that were not included in training. This is important because model performance can appear better when closely related compounds are present in both the training and test sets. For this reason, validation strategies that reduce such overlap are considered more appropriate when the aim is to evaluate performance for previously unseen compounds [46,47]. Accordingly, the present comparison should be understood as a stricter test of whether machine-learning correction provides added value beyond the baseline COSMO-RS model.

The implication is clear in that the value of machine learning in solubility prediction is not determined by dataset diversity per se but rather by the alignment between the chemical structure of the solute and the theoretical framework. For compounds whose solvation behavior is dominated by interactions that COSMO-RS handles well, such as hydrogen bonding in phenolic acids, theory alone is sufficient. For compounds with more complex or varied interaction landscapes, such as active pharmaceutical ingredients spanning multiple chemical classes, machine learning can identify correction patterns that theoretical models overlook. This insight reframes the common narrative. Rather than viewing machine learning as a universal improvement over theory, it should be understood as a complementary tool whose value depends on the chemical characteristics of the target compounds.

### 2.5. Error Structure and Applicability Domain

**Figure 5:**
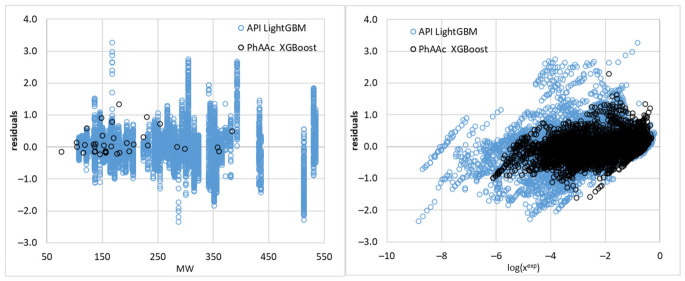
Residual analysis of DOOIT2 model predictions. (**left**) Residuals (predicted − experimental log mole fraction solubility) plotted as a function of molecular weight (MW). Residuals plotted as a function of experimental solubility (log x_exp) (**right**). Blue points correspond to the API dataset (LightGBM model), and orange points correspond to the PhAAc dataset (XGBoost model). The plots illustrate the distribution of prediction errors across molecular size and solubility range, highlighting increased variability in the low-solubility region and the absence of a clear dependence of residuals on molecular weight.

To better understand the predictive behavior of the model, the distribution of residuals was analyzed as a function of both molecular properties and experimental solubility. As shown in the left panel of Figure 5, the residuals plotted against molecular weight do not exhibit a clear systematic trend. Although a slight increase in dispersion can be observed for higher molecular weights, the relationship is not sufficiently pronounced to support a direct dependence of prediction error on molecular size alone. This indicates that molecular weight, as an isolated descriptor, is not a primary determinant of model performance. In contrast, a more distinct pattern emerges when residuals are analyzed as a function of experimental solubility, as shown in the right panel of Figure 5. The model exhibits larger variability in the low-solubility region (more negative log(x)), whereas predictions become more tightly distributed at higher solubility values. This behavior reflects the increased difficulty of accurately predicting very low solubility, where small absolute deviations correspond to larger errors on the logarithmic scale.

These observations suggest that the primary limitation of the model is associated with the intrinsic difficulty of predicting low-solubility systems rather than with simple molecular descriptors such as size or flexibility. Consequently, the applicability domain of the model should be interpreted in terms of solubility regime and data representation rather than strictly structural parameters. The residual analysis indicates that the model performs consistently across a wide range of compounds, with increased uncertainty primarily confined to the low-solubility region.

### 2.6. The Role of 4-Formylmorpholine

The role of 4-formylmorpholine (4FM) in the present study was primarily to provide an experimentally characterized yet underrepresented solvent environment for testing model transferability. The newly measured 4FM-water systems therefore complement the broader literature-derived datasets by extending them toward a solvent that is less commonly represented in pharmaceutical solubility studies. For these systems, the prediction errors remained consistent with the broader behavior observed for the respective datasets and did not indicate any unusual deterioration associated with 4FM. This, in turn, suggests that the molecular features of 4FM are represented sufficiently well in the descriptor space to permit interpolation despite its more limited representation in the training data. Thus, beyond expanding the experimental knowledge base for 4FM as a pharmaceutical co-solvent, these measurements also provide a practical test of model transferability to a solvent system that bridges amide- and ether-like chemical features.

### 2.7. Limitations and Future Directions

Several limitations of this study should be acknowledged. First, although the training set for the API dataset is diverse and contains 85 compounds, it remains limited relative to the full chemical space of pharmaceutical compounds. The residual analysis indicates that prediction uncertainty is most pronounced in the low-solubility region, and further expansion of the dataset toward underrepresented structural classes would improve model robustness. Second, while the PhAAc dataset covers 57 solvent systems, it remains a chemically narrower acid-centered dataset. Extending this dataset to additional acidic compound families would help determine whether the present findings generalize beyond the current chemical domain. Third, the current descriptor set does not explicitly account for solute ionization, which may be relevant for ionizable APIs in aqueous mixtures. The incorporation of ionization descriptors, including pKa, speciation, and pH-dependent solubility, could improve predictions for compounds whose solubility is strongly affected by protonation state.

Future work will focus on several key directions. Initial efforts will target the strategic expansion of the API dataset to include more compounds from underperforming classes. This involves adding large and flexible molecules like peptides and macrocycles alongside compounds with basic nitrogen functionalities to fill gaps in the chemical space. Subsequent efforts will extend the PhAAc dataset to include other carboxylic acid families, which will enable an assessment of whether the findings for phenolic acids generalize to aliphatic and dicarboxylic acids. Another objective involves the integration of ionization descriptors to capture pH-dependent solubility effects that are critical for many APIs. Additionally, the transferability of the DOOIT2 framework to other properties such as permeability, stability, and dissolution rate, as well as to other solvent systems like alcohols, polyols, and lipid-based excipients, will be explored in subsequent studies.

## 3. Materials and Methods

### 3.1. Materials

Vanillic acid (97%, CAS 121-34-6), 4-formylmorpholine (99%, CAS 4394-85-8), lidocaine (≥98%, CAS 137-58-6), and benzocaine (≥99%, CAS 94-09-7) were obtained from Sigma-Aldrich (St. Louis, MO, USA). Analytical-grade methanol was purchased from Pol-Aura (Morąg, Poland).

### 3.2. Experimental Determination of Solute Solubility

Solubility was determined using a shake-flask procedure adapted for binary 4FM-water media. The investigated solvent compositions covered the entire composition range of the solute-free binary mixture, from pure water to pure 4FM, with intermediate 4FM mole fractions spaced by 0.1. For each composition, an excess amount of the appropriate solid was introduced into test tubes containing the pre-prepared solvent mixture. The suspensions were equilibrated for 24 h at 298.15 K in an ES-20/60 Orbital Shaker Incubator (Biosan, Riga, Latvia) operated at 60 rpm. The temperature setting accuracy was 0.1 °C, and the variation during the equilibration period did not exceed 0.5 °C.

After incubation, the saturated samples were filtered through PTFE syringe filters with a pore size of 0.22 µm. To reduce the risk of precipitation during sample handling, all accessories coming into contact with the equilibrated solutions, including test tubes, pipette tips, syringes, and filters, were pre-equilibrated at the same temperature as the samples before filtration. Appropriate aliquots of the clear filtrates were then analyzed spectrophotometrically. Three independent saturated samples were prepared and measured for each solvent composition.

Quantification was based on individual calibration curves prepared separately for vanillic acid, benzocaine, and lidocaine. The calibration ranges were 0.00090–0.06370 mg/mL for vanillic acid, 0.00066–0.01340 mg/mL for benzocaine, and 0.02137–0.68384 mg/mL for lidocaine. Absorbance was measured at 295 nm for vanillic acid, 292 nm for benzocaine, and 263 nm for lidocaine using an A360 spectrophotometer (AOE Instruments, Shanghai, China). The corresponding linear calibration equations were A = 33.4789C − 0.0265 for vanillic acid, A = 123.8788C − 0.0201 for benzocaine, and A = 1.4853C − 0.0031 for lidocaine. In all cases, the coefficient of determination (*R*^2^) was 0.999. Here, A denotes absorbance and C is the concentration expressed in mg/mL. Each calibration point represented the mean of three measurements. The limits of detection (LOD) and quantification (LOQ), calculated as 3.3σ/S and 10σ/S, respectively, were 0.00010 and 0.00029 mg/mL for vanillic acid, 0.00018 and 0.00056 mg/mL for benzocaine, and 0.00326 and 0.00987 mg/mL for lidocaine.

The density of each saturated solution was determined gravimetrically by weighing 1 mL aliquots transferred with an Eppendorf Reference 2 pipette (Eppendorf AG, Hamburg, Germany) into 10 mL volumetric flasks. The systematic pipette error was 6 μL. Mass measurements were performed using a RADWAG AS 110.R2 PLUS analytical balance (RADWAG, Radom, Poland) with a readability of 0.1 mg. The concentrations determined from the UV-Vis measurements were subsequently converted into mole fraction solubilities using the experimentally determined solution densities and the molar masses of the solute, 4FM, and water according to Equation (1):

(1) $$X^{Solute}=\frac{\frac{C^{Solute}}{M^{Solute}}}{\frac{C^{Solute}}{M^{Solute}}+\frac{1000\cdot \rho -C^{Solute}}{x_{4FM}^{\prime }M_{4FM}+x_{H2O}^{\prime }M_{H2O}}}$$

where *X^Solute^* is the mole fraction solubility of the solute, *C^Solute^* is the solute concentration expressed in mg/mL, ρ is the density of the saturated solution expressed in g/mL, *M^Solute^*, *M*_4*FM*_, and *M_H_*_2*O*_ are the molar masses of the solute, 4FM, and water, respectively, and *x*′_4*FM*_ and *x*′*_H_*_2*O*_ denote the mole fractions of 4FM and water in the solute-free binary solvent mixture. All experiments were performed in triplicate.

### 3.3. COSMO-RS Computations

Quantum-chemical calculations were performed using COSMO-RS theory as implemented in COSMOtherm (version 2024, COSMOlogic GmbH & Co. KG, Leverkusen, Germany). For each solute and solvent, geometry optimizations were carried out at the BP86/TZVP level of theory using the TURBOMOLE (version 7.8) program package. The resulting COSMO files were used to generate σ-profiles and σ-potentials. Prior to COSMO calculations, all structures were fully geometry-optimized at the BP86/TZVP level of theory, ensuring proper pre-optimization before σ-profile generation.

Solubility predictions were obtained using the reference solvent approach, which anchors the calculation to experimental solubility values in neat solvents. For each solute, the experimental solubility in pure water and pure organic solvent at the relevant temperature was supplied as input. The computed solubility in binary mixtures was then obtained via interpolation based on COSMO-RS interaction energies. This approach effectively removes the error associated with solute fusion properties and focuses the prediction entirely on mixture effects.

A comprehensive set of molecular descriptors was extracted from the COSMO-RS output and used as model inputs in the DOOIT2 workflow, whereas the prediction target was the experimental decadal logarithm of mole fraction solubility, log(x_exp_). The baseline theoretical predictor was RefSol, i.e., the COSMO-RS decadal logarithm of mole fraction solubility obtained with the reference-solvent approach. As summarized in Table 2, Set 1 comprised energetic and chemical-potential descriptors derived from COSMO-RS for the solute, the solvent mixture, and their relative differences under saturated conditions.

Set 2 extended this representation by adding the σ-potential descriptor block. The standard COSMO-RS σ-profile, comprising 61 data points across a charge density range from –0.03 to +0.03 e·Å^−2^, was condensed by averaging the values over intervals of 0.005 e·Å^−2^. This produced a 12-step function covering the hydrogen bond donor (HBD), hydrophobic (HH), and hydrogen bond acceptor (HBA) regions. Descriptors were then defined for the solute, the solvent mixture, and their relative differences, as detailed in Supplementary Tables S3.1 and S3.2.

Set 3 does not represent a separate descriptor-generation step. Instead, it denoted the reduced feature configuration retained after iterative pruning and model selection within the DOOIT2 procedure. Accordingly, the final PhAAc model was selected from the Set 2 representation, whereas the final API model was selected from the model trained on features selected from Set 2 and reduced to Set 3 during feature selection.

### 3.4. Datasets

Two complementary datasets were used in this study, and both were curated with explicit source tracking at the level of the solute-solvent pair. Details of the content are provided in the Supporting Materials (see Section S2). This procedure was introduced to standardize compound names, harmonize solvent labels, identify duplicated or order-inverted solvent-pair entries across literature sources, and resolve obvious bibliographic formatting inconsistencies while preserving transparent provenance for every record retained in the final descriptor table used for model development. The division into the API and PhAAc datasets was operational and based on the intended chemical scope of each set rather than on a formal external taxonomy. The API dataset was designed to maximize structural diversity and therefore grouped active pharmaceutical ingredients together with related bioactive or formulation-relevant solutes measured mainly in aqueous mixtures with polar aprotic and related organic cosolvents. In contrast, the PhAAc dataset was designed as a chemically narrower acid-centered set composed mainly of phenolic acids and related carboxylic acids, while also retaining selected heteroaromatic, dicarboxylic, amino-, and hydroxy-acids that preserved this broader acidic chemical domain. The final API dataset comprised 85 unique solutes and 10,140 solubility records, whereas the final PhAAc dataset comprised 37 unique solutes and 6030 solubility records. In both datasets, the full composition range between the two neat components of a given binary system was retained whenever available, and records obtained at comparable temperatures were merged only after consistency checking. For transparency, the temperature range covered for each solute–solvent system is provided in Supplementary Tables S2.1 and S2.2. The complete pair-level provenance was preserved in the curated workbook used for descriptor generation and model development.

The PhAAc dataset was assembled from literature data for phenolic acids, substituted benzoic and cinnamic acids, heteroaromatic and dicarboxylic acids, amino- and hydroxy-acids, and selected acidic drug-like compounds. In addition to classical phenolic and benzoic-acid derivatives, this dataset also covered compounds such as mesalazine, ibuprofen, naproxen, ketoprofen, indomethacin, artesunate, zaltoprofen, d-histidine, gamma-aminobutyric acid, 4-aminobutyric acid, hydroxyacetic acid, succinic acid, malonic acid, maleic acid, adipic acid, and related systems [48,49,50,51,52,53,54,55,56,57,58,59,60,61,62,63,64,65,66,67,68,69,70,71,72,73,74,75,76,77,78,79,80,81,82,83,84,85,86,87,88,89,90,91,92,93,94,95,96,97,98,99,100,101,102,103,104,105,106,107]. Furthermore, this dataset incorporates the new experimental measurements for vanillic acid in mixtures of 4-formylmorpholine and water reported in the present study.

The API dataset was assembled to maximize structural diversity and included anti-infective agents, sulfonamides and related antimicrobial compounds, xanthines and related polar heterocycles, aromatic amides and analgesic-type compounds, local anesthetics, nitro- and halo-substituted aromatic compounds, triazoles and other N-heterocycles, neutral bioactive compounds, sugars and polyol-related compounds, and several other formulation-relevant solutes retained in the curated workbook. This set included, among others, allopurinol, dapsone, sulfadiazine, sulfamethazine, sulfamethizole, sulfamethoxazole, sulfamethoxypyridazine, sulfapyridine, sulfisomidine, sulfanilamide, carbendazim, ketoconazole, clotrimazole, lamotrigine, maraviroc, ribavirin, acipimox, amrinone, acetanilide, paracetamol, phenacetin, caffeine, theobromine, theophylline, benzamide, benzenesulfonamide, salicylamide, ethenzamide, griseofulvin, gliclazide, isotretinoin, triclocarban, celecoxib, coumarin, chrysin, naringenin, salicin, l-fucose, adenosine, doxofylline, d-histidine, and several nitroaromatic or agrochemical-like compounds [9,54,58,60,68,74,81,87,91,96,98,107,108,109,110,111,112,113,114,115,116,117,118,119,120,121,122,123,124,125,126,127,128,129,130,131,132,133,134,135,136,137,138,139,140,141,142,143,144,145,146,147,148,149,150,151,152,153,154,155,156,157,158,159,160,161,162,163,164,165,166,167,168,169,170,171,172,173,174,175,176,177,178,179,180,181,182]. Furthermore, this dataset incorporates the new experimental measurements for lidocaine and benzocaine in mixtures of 4-formylmorpholine and water reported in the present study.

### 3.5. Machine Learning Framework: DOOIT2

Predictive models were developed using the DOOIT2 framework, which integrates feature selection, hyperparameter optimization, and model validation into a unified workflow. DOOIT2 represents a significant methodological advancement over our previously published DOOIT framework [23,45,74] by transitioning from repeated random train-test splits to API-out Structured Group K-Fold (SGKF) validation combined with fold-specific ensemble models.

#### 3.5.1. Core Algorithms and Data Preprocessing

Based on preliminary benchmarking, LightGBM (Light Gradient Boosting Machine, version 4.6.0) was selected as the core regressor for the APIs dataset, whereas XGBoost (Extreme Gradient Boosting, version 3.2.0) was employed for the PhAAc dataset. Both algorithms are tree-based ensemble methods capable of capturing complex and non-linear relationships while maintaining interpretability through feature importance metrics. Prior to model training, all descriptors were standardized by removing the mean and scaling to unit variance using the StandardScaler from the scikit-learn library (version 1.7.2). This procedure ensures that no single descriptor disproportionately influences the model due to differences in scale.

#### 3.5.2. API-Out Validation with Fold-Specific Ensemble Models

To rigorously assess generalization to unseen chemical entities, all models were validated using API-out SGKF cross-validation. In this scheme, the dataset was partitioned by solute identity to ensure that all measurements for a given compound were assigned exclusively to either the training or the validation fold. For the APIs dataset, 85 unique solutes were distributed across five folds, with each fold holding out approximately 17 APIs for validation. For the PhAAc dataset, 37 solutes were similarly distributed across five folds.

Crucially, DOOIT2 adopts a fold-specific ensemble modeling approach. Rather than producing a single champion model trained on a fixed training set, the framework independently develops a separate model for each SGKF fold. Each fold-specific model is trained exclusively on data from the remaining four folds, utilizing all solutes except those assigned to the held-out fold, and is optimized to predict solubility for the compounds in its corresponding held-out fold. The result is an ensemble of five models, each with its own potentially distinct feature set and hyperparameters. For any given compound, the prediction is made by the specific fold model for which that compound was held out during training. This ensures that every prediction is genuinely out-of-sample with respect to compound identity.

#### 3.5.3. Dual-Objective Optimization and Iterative Feature Pruning

Model optimization was formulated as a dual-objective problem balancing predictive accuracy against model complexity. These objectives included the minimization of the Mean Absolute Error (MAE) evaluated via 5-fold cross-validation alongside the minimization of model complexity. For tree-based models, this complexity was quantified as the total number of trees multiplied by the average tree depth. Optimization was performed using the Optuna framework (version 3.2), utilizing the Tree-structured Parzen Estimator (TPE) sampler. For each independent run, 2000 optimization trials were conducted to ensure a comprehensive exploration of the hyperparameter space. This process yielded a Pareto front of non-dominated solutions that represent the best achievable trade-off between accuracy and complexity.

Feature selection was integrated into the optimization workflow through iterative backward pruning. Starting with the full descriptor set, a specific sequence of steps was repeated until a minimum feature count was reached. First, dual-objective optimization was performed on the current feature set. A candidate model was then selected from the Pareto front using the one-standard-error (1-SE) rule. This rule identifies the simplest model whose cross-validated MAE falls within one standard error of the best-performing model. Following this selection, feature importance was computed using permutation importance with 10 repetitions, and the least impactful feature was subsequently eliminated. This iterative process generated a family of candidate models at each level of complexity.

#### 3.5.4. Model Selection and Stability Analysis

To ensure the selection of a robust and reproducible model, the entire DOOIT2 procedure was repeated 15 independent times using a different random seed for data partitioning and optimization in each iteration. A multi-criteria selection framework was subsequently applied to identify the final champion model. Architectural stability was assessed by requiring descriptor counts to appear in at least 30% of the independent runs, and the optimal descriptor count was identified as the simplest architecture meeting this threshold. From the architecturally stable group, a specific model instance was selected using a composite scoring system that balanced predictive accuracy with a 50% weight, explanatory power measured by *R*^2^ with a 30% weight, and generalization stability based on the train-test performance gap with a 20% weight. The final selected models, specifically LightGBM for the APIs dataset and XGBoost for the PhAAc dataset, were subjected to comprehensive residual analysis and applicability domain assessment to confirm their reliability.

#### 3.5.5. Methodological Advantages

The transition to API-out SGKF with fold-specific ensemble models provides three key benefits. First, it delivers enhanced methodological validity. By enforcing chemical separation at the solute level, DOOIT2 provides a more conservative but fundamentally more honest estimate of predictive power for novel compounds than random split validation. Second, it improves robustness and stability analysis. Evaluating feature stability across fold-specific models ensures that the selected descriptors reflect generalizable physicochemical relationships rather than dataset-specific artifacts. The stability metric of 0.909 for the APIs dataset directly quantifies this reproducibility. Third, it explicitly aligns with real-world applications. Formulation scientists encounter new compounds rather than randomly selected data points, and DOOIT2 validates models under conditions that mirror this reality.

#### 3.5.6. Implementation

The DOOIT2 framework was implemented as a fully automated pipeline in Python 3.10 utilizing the scikit-learn library (version 1.3) for preprocessing and model evaluation, Optuna (version 3.2) for hyperparameter optimization, and pandas (version 2.0) for data management. For each dataset, the pipeline was executed with a 5-fold SGKF to generate five-fold-specific models. The complete code, which includes the implementation of the fold-specific ensemble logic, and the data necessary to reproduce all results are provided in the Supplementary Materials.

### 3.6. Descriptor Characterization

In line with the methodology detailed in our prior study [23,45], all molecular descriptors were computed from first-principles data using the COSMO-RS (Conductor-like Screening Model for Real Solvents) approach [183,184,185]. The workflow for generating these descriptors proceeds through three defined phases. Initially, a conformational analysis is conducted, involving extensive conformer searches for each solute and solvent molecule via COSMOconf under default settings. To adequately map the conformational space, no more than ten of the most stable conformers are kept for follow-up thermodynamic analysis. Geometry refinement and COSMO output file creation are then performed using TURBOMOLE [186] at the RI-BP/TZVP level, with the TZVPD-FINE basis set (BP_TZVPD_FINE_24.ctd) as per established practices [20,187,188]. Subsequently, the COSMO and energy files required by COSMOtherm [189] to determine interaction energies and chemical potentials are assembled for each solute–solvent system considered in this study. The final mixture calculation outputs provide the descriptors used in the present machine-learning workflow. Their definitions are summarized in Supplementary Tables S3.1 and S3.2.

Apart from energetic and chemical-potential descriptors, the final set of molecular descriptors was augmented with values derived from σ-potential distributions. The standard COSMO-RS output consists of 61 data points covering the charge density range of −0.03e/Å^2^ + 0.03e/Å^2^. Consistent with prior machine learning applications, this data was reduced by averaging values over 0.005 intervals. This process resulted in a 12-step function defining three characteristic regions of the σ-potential: hydrogen bond donor (HBD1-4, −0.03e/Å^2^ to −0.01e/Å^2^), hydrophobicity, (HH1-4, from −0.01e/Å^2^ to +0.01e/Å^2^), and acceptability (Hydrogen Bond Acceptor, HBA1-4, from +0.01e/Å^2^ to +0.03e/Å^2^). Consequently, four descriptors were generated for each region, leading to 24 descriptors of this type for the solute, the solvent, and the relative difference between them (Supplementary Table S3.2).

### 3.7. Performance Metrics

Model performance was evaluated using three complementary metrics describing predictive accuracy, explanatory power, and model reproducibility. The root mean square deviation (*RMSD*) was used as the primary measure of prediction error, the coefficient of determination (*R*^2^) was used to quantify the proportion of variance explained by the model, and a stability metric was used to assess the reproducibility of descriptor selection across independent optimization runs.

The root mean square deviation (*RMSD*) was calculated as follows:

(2) $$RMSD=\sqrt{\frac{1}{n}\sum_{i=1}^{n}({y_{i}-{\hat{y}}_{i})}^{2}}$$

where *y_i_* and *ŷ_i_* are the experimental and predicted log mole fraction solubilities, respectively, and *n* is the number of observations.

The coefficient of determination (*R*^2^) was calculated as follows:

(3) $$R^{2}=1-\frac{\sum_{i=1}^{n}{\left(y_{i}-{\hat{y}}_{i}\right)}^{2}}{\sum_{i=1}^{n}{\left(y_{i}-\bar{y}\right)}^{2}}$$

To complement the predictive metrics, model stability for the APIs dataset was quantified as the average similarity of selected feature sets across independent runs, where values approaching 1.0 indicate high reproducibility. For the final API model selected in this study, the stability metric was 0.909. The stability metric was defined as follows:

(4) $$Stability=\frac{2}{k(k-1)}\sum_{i<j}\frac{\left|F_{i}\cap \;F_{j}\right|}{\left|F_{i}\cup F_{j}\right|}$$

where *F_i_* and *F_j_* represent the feature sets selected in independent runs *i* and *j*, and *k* denotes the total number of runs. All reported performance metrics are based on API-out SGKF cross-validation to ensure that the results reflect true generalization to unseen compounds.

## 4. Conclusions

This study provides a systematic evaluation of when machine learning adds value beyond COSMO-RS for predicting API solubility in binary mixtures. The results demonstrate that COSMO-RS performs well for chemically homogeneous systems, such as phenolic and carboxylic acids, where solvation behavior is governed by well-defined interaction patterns. In contrast, for structurally diverse APIs, the hybrid DOOIT2 framework provides improved predictive performance, although the magnitude of improvement remains moderate.

Importantly, these findings show that the benefit of machine learning is not universal but depends on the chemical diversity of the solute space and the complexity of solvation interactions. The use of API-out validation highlights that even modest improvements under strict generalization conditions are meaningful. At the same time, residual analysis indicates that prediction uncertainty increases in the low-solubility regime, defining a practical applicability domain.

These results therefore support a complementary strategy in which machine learning is applied selectively, as a refinement of physics-based models, rather than as a universal replacement.

## Figures and Tables

**Table 1:** Comparative Feature Selection of DOOIT2 Models for PhAAc and API Datasets.

Descriptor Category	PhAAc (XGBoost)	API (LightGBM)	Key Insight
Baseline	RefSol (100%)	RefSol (100%)	Both rely on COSMO-RS foundation
Solute-solvent differences	Low (20–40%)	High (80%)	Critical for diverse APIs
Hydrogen bond donor	Moderate (40%)	Very High (100%)	Donor capacity varies widely in APIs
Hydrophobic region	d_HH2, d_HH3 (80–100%)	d_HH1 (100%)	Different hydrophobic bins dominate
Solute van der Waals	Moderate (60%)	Very High (100%)	Universal importance for APIs
Solvent descriptors	High (60%)	Low (20%)	API model focuses on solute variability

**Table 2:** Summary of target variables, model inputs, and feature configurations used in the DOOIT2 workflow.

Item	Role in the Workflow	Description
log(x_exp_)	Prediction target (output)	Experimental decadal logarithm of mole fraction solubility
RefSol	Baseline input	COSMO-RS predicted decadal logarithm of mole fraction solubility obtained with the reference-solvent approach
Set 1	Descriptor configuration	Energetic and chemical-potential descriptors derived from COSMO-RS for the solute, solvent mixture, and their relative differences
Set 2	Expanded descriptor configuration	Set 1 extended with the σ-potential descriptor block covering HBD, HH, and HBA regions for the solute, solvent mixture, and their relative differences
Set 3	Final reduced feature configuration	Reduced feature set retained after iterative pruning and model selection within DOOIT2; used for the final API model

## Data Availability

The original contributions presented in this study are included in the article/Supplementary Materials. Further inquiries can be directed to the corresponding authors.

## Supplementary Materials

The following supporting information can be downloaded at: https://www.mdpi.com/article/10.3390/molecules31101566/s1, Table S1.1: Newly determined experimental solubility data for lidocaine, benzocaine, and vanillic acid in binary 4-formylmorpholine (4FM)-water mixtures at 298.15 K, obtained in this study. Solubility is expressed as C^solute^ (mg/mL) and x^Solute^ as a function of the mole fraction of 4FM in the solute-free mixed solvent (x_4FM_). SD, standard deviation; Table S2.1: Characteristics of the API dataset used for model development. N denotes the number of solubility records taken from the cited source, and temperature range refers to the experimental temperature range covered by the corresponding solute–solvent system; Table S2.2: Characteristics of the PhAAc dataset used for model development. N denotes the number of solubility records taken from the cited source, and temperature range refers to the experimental temperature range covered by the corresponding solute–solvent system; Figure S2.1: Dataset diversity illustrated by the relationship between hydrogen-bond donor character (HBD) and (a) hydrogen-bond acceptor character (HBA) or (b) hydrophobic character (HH) for the API and PhAAc datasets. HBD = HBD1 + HBD2 + HBD3 + HBD4, HBA = HBA1 + HBA2 + HBA3 + HBA4, and HH = HH1 + HH2 + HH3 + HH4; Table S3.1: Detailed explanations of the descriptors used in the study. The acronyms are consistent with the terminology used in the spreadsheet in the Supplementary Materials; Table S3.2: Detailed explanations of the σ-potential related descriptors used in the study. The acronyms are consistent with the terminology used in the spreadsheet in the Supplementary Materials; Table S4.1: Fold-wise feature selection results across SGKF folds for the PhAAc dataset (XGBoost); Table S4.2: Fold-wise feature selection results across SGKF folds for the API dataset (LightGBM).
