# Supplementary material for: When Does Machine Learning Add Value over Theory? Predicting API Solubility in Binary Mixtures with COSMO-RS and DOOIT2 Across Diverse and Homogeneous Systems
Source: Molecules. 2026 May 8;31(10):1566. doi: 10.3390/molecules31101566 (PMC13210068; doi:10.3390/molecules31101566)
Supplement: Supplementary file 1 [file molecules-31-01566-s001.zip › molecules-4262438-supplementary.pdf]

Supplementary Materials

S1. Experimental results 2

Table S1.1 Newly determined experimental solubility data for lidocaine, benzocaine, and vanillic acid in binary 4-formylmorpholine (4FM)-water mixtures at 298.15 K, obtained in this study. Solubility is expressed as C^{solute} (mg/mL) and x^{Solute} as a function of the mole fraction of 4FM in the solute-free mixed solvent (x^{4FM}). SD, standard deviation. 2

S2. Dataset 3

Table S2.1. Characteristics of the API dataset used for model development. N denotes the number of solubility records taken from the cited source, and temperature range refers to the experimental temperature range covered by the corresponding solute–solvent system. 3

Table S2.2. Characteristics of the PhAAc dataset used for model development. N denotes the number of solubility records taken from the cited source, and temperature range refers to the experimental temperature range covered by the corresponding solute–solvent system. 5

Figure S2.1. Dataset diversity illustrated by the relationship between hydrogen-bond donor character (HBD) and (a) hydrogen-bond acceptor character (HBA) or (b) hydrophobic character (HH) for the API and PhAAc datasets. HBD = HBD1 + HBD2 + HBD3 + HBD4, HBA = HBA1 + HBA2 + HBA3 + HBA4, and HH = HH1 + HH2 + HH3 + HH4. 6

S3. Descriptors used for ML 7

Table S3.1. Detailed explanations of the descriptors used in the study. The acronyms are consistent with the terminology used in the spreadsheet in the Supplementary Materials. 7

Table S3.2. Detailed explanations of the σ-potential related descriptors used in the study. The acronyms are consistent with the terminology used in the spreadsheet in the Supplementary Materials. 7

S4. Feature Selection Across SGKF Folds 9

Table S4.1. Fold-wise feature selection results across SGKF folds for the PhAAc dataset (XGBoost). 9

Table S4.2. Fold-wise feature selection results across SGKF folds for the API dataset (LightGBM) 10

S1. Experimental results

Table S1.1 Newly determined experimental solubility data for lidocaine, benzocaine, and vanillic acid in binary 4-formylmorpholine (4FM)-water mixtures at 298.15 K, obtained in this study. Solubility is expressed as C^{solute} (mg/mL) and x^{Solute} as a function of the mole fraction of 4FM in the solute-free mixed solvent (x^{4FM}). SD, standard deviation.

x^{4FM}	C^{Solute} [mg/ml]	SD	x^{Solute} × 100	SD × 100
**Lidocaine**				
0.0	4.040	0.131	0.031	0.001
0.1	36.466	1.127	0.413	0.013
0.2	85.329	0.988	1.331	0.016
0.3	164.175	9.408	3.402	0.220
0.4	257.341	18.459	6.918	0.608
0.5	324.046	1.398	10.634	0.058
0.6	371.538	16.544	14.201	0.824
0.7	425.397	5.587	18.443	0.319
0.8	472.983	13.848	23.382	0.913
0.9	526.617	15.161	28.769	1.117
1.0	555.817	6.958	32.987	0.553
**Benzocaine**				
0.0	0.966	0.047	0.011	0.001
0.1	35.801	1.167	0.571	0.019
0.2	124.507	6.007	2.765	0.146
0.3	194.886	10.552	5.612	0.345
0.4	276.245	9.109	10.051	0.394
0.5	334.306	14.806	14.328	0.769
0.6	386.369	9.562	19.187	0.581
0.7	424.240	12.921	23.577	0.871
0.8	456.499	18.579	27.752	1.363
0.9	478.107	14.806	31.116	1.131
1.0	479.879	1.061	33.581	0.085
**Vanillic acid**				
0.0	2.709	0.104	0.029	0.001
0.1	48.917	1.242	0.775	0.020
0.2	132.366	4.557	2.864	0.109
0.3	198.873	6.787	5.508	0.214
0.4	244.570	12.718	8.087	0.487
0.5	283.776	15.205	10.872	0.677
0.6	313.218	14.591	13.706	0.745
0.7	331.866	12.966	16.272	0.735
0.8	347.358	12.093	18.724	0.746
0.9	350.912	3.406	20.212	0.220
1.0	340.342	0.949	20.943	0.064

S2. Dataset

Table S2.1. Characteristics of the API dataset used for model development. N denotes the number of solubility records taken from the cited source, and temperature range refers to the experimental temperature range covered by the corresponding solute–solvent system.

Solute	Solvent 1	Solvent 2	N	Temperature range [K]	Source
allopurinol	1-methyl-2-pyrrolidone	water	99	293.15–333.15	https://doi.org/10.1016/j.jct.2018.11.028
5,7-Dibromo-8-hydroxyquinoline	water	NMP	110	288.15–333.15	https://doi.org/10.1016/j.jct.2020.106138
griseofulvin	water	NMP	110	278.15–323.15	https://doi.org/10.1016/j.jct.2020.106250
d-Histidine	water	NMP	99	293.15–333.15	https://pubs.acs.org/doi/10.1021/acs.jced.9b01051
ketoconazole	water	N-methyl-2-pyrrolidone	55	293.20–313.20	https://doi.org/10.1016/j.molliq.2019.02.038
adenosine	NMP	water	110	278.15–323.15	https://doi.org/10.1016/j.jct.2017.07.023
amoxicillin	water	N-methyl pyrrolidone	121	278.15–328.15	https://doi.org/10.1016/j.jct.2019.106010
rivaroxaban	NMP	water	66	273.15–323.15	https://pubs.acs.org/doi/10.1021/acs.jced.5b00667
5-aminosalicylic acid	N-methyl-2-pyrrolidone	water	55	293.20–313.20	https://doi.org/10.1016/j.molliq.2020.113143
Terephthalaldehydic acid	NMP	water	99	283.15–323.15	https://pubs.acs.org/doi/10.1021/acs.jced.8b01262
celecoxib	N-methyl-2-pyrrolidone	water	55	293.20–313.20	https://doi.org/10.1007/s11814-017-0028-y
Sulfadiazine	N-methyl-2-pyrrolidone	water	88	278.15–313.15	https://doi.org/10.1016/j.molliq.2021.115693
triclocarban	N-Methyl-2-pyrrolidone	water	147	288.15–318.15	https://doi.org/10.3390/molecules28207216
benzamide	4-formylmorpholine	water	24	298.15–313.15	https://doi.org/10.3390/molecules27103323
salicylamide	4-formylmorpholine	water	24	298.15–313.15	https://doi.org/10.3390/molecules27103323
ethenzamide	4-formylmorpholine	water	24	298.15–313.15	https://doi.org/10.3390/molecules27103323
Benzenesulfonamide	4-Formylmorpholine	water	24	298.15–313.15	https://doi.org/10.3390/molecules28135008
Paracetamol	4-Formylmorpholine	water	24	298.15–313.15	https://doi.org/10.3390/pharmaceutics14122828
Phenacetin	4-Formylmorpholine	water	24	298.15–313.15	https://doi.org/10.3390/pharmaceutics14122828
Sulfamethizole	4-Formylmorpholine	water	24	298.15–313.15	https://apd.umk.pl/diplomas/138379/
Sulfamethoxazole	4-formylmorpholine	water	24	298.15–313.15	https://doi.org/10.3390/molecules29204894
Sulfanilamide	4-formylmorpholine	water	24	298.15–313.15	https://polimery.umw.edu.pl/en/article/2024/54/1/27/
sulfathiazole	4-formylmorpholine	water	24	298.15–313.15	https://apd.umk.pl/diplomas/138379/
caffeic acid	4-formylmorpholine	Water	11	298.15	https://doi.org/10.3390/molecules30224444
Ferulic acid	4-formylmorpholine	Water	11	298.15	https://doi.org/10.3390/molecules30224444
lidocaine	4-formylmorpholine	water	11	298.15	This work
benzocaine	4-formylmorpholine	water	11	298.15	This work
vanillic acid	4-formylmorpholine	water	11	298.15	This work
allopurinol	DMF	water	99	293.15–333.15	https://doi.org/10.1016/j.jct.2018.11.028
5,7-Dibromo-8-hydroxyquinoline	water	DMF	110	288.15–333.15	https://doi.org/10.1016/j.jct.2020.106138
griseofulvin	water	DMF	110	278.15–323.15	https://doi.org/10.1016/j.jct.2020.106250
d-Histidine	water	DMF	99	293.15–333.15	https://pubs.acs.org/doi/10.1021/acs.jced.9b01051
Sulfadiazine	DMF	water	30	293.15–313.15	https://doi.org/10.1111/j.2042-7158.1968.tb09656.x
Amrinone	water	DMF	110	278.15–323.15	https://pubs.acs.org/doi/10.1021/acs.jced.0c00393
4-Nitrophthalimide	water	DMF	110	278.15–323.15	https://pubs.acs.org/doi/10.1021/acs.jced.0c00479
carbendazim	DMF	water	99	278.15–318.15	https://doi.org/10.1016/j.jct.2018.08.001
adenosine	DMF	water	110	278.15–323.15	https://doi.org/10.1016/j.jct.2017.07.023
buprofezin	DMF	water	110	273.15–318.15	https://doi.org/10.1016/j.jct.2019.06.019
gimeracil	water	DMF	110	283.15–328.15	https://doi.org/10.1016/j.jct.2019.01.026
amoxicillin	water	DMF	121	278.15–328.15	https://doi.org/10.1016/j.jct.2019.106010
D-tryptophan	water	DMF	121	283.15–333.15	https://doi.org/10.1016/j.jct.2018.08.018
3-Methyl-6-nitroindazole	DMF	water	88	278.15–328.15	https://pubs.acs.org/doi/10.1021/acs.jced.8b01256
chrysin	DMF	Water	11	298.15	https://doi.org/10.1016/j.molliq.2016.05.019
2-Aminobenzoic acid	DMF	Water	30	288.18–308.15	https://doi.org/10.1016/j.molliq.2020.112566
meloxicam	DMF	water	55	293.15–313.15	https://doi.org/10.1016/j.jct.2020.106332
maraviroc	DMF	Water	110	278.15–323.15	https://doi.org/10.1016/j.jct.2019.106044
Thiamphenicol	DMF	Water	99	278.15–318.15	https://pubs.acs.org/doi/10.1021/acs.jced.8b00179
gliclazide	DMF	water	95	278.15–318.15	https://doi.org/10.1016/j.molliq.2020.113258; https://doi.org/10.1016/j.molliq.2020.113425
sulfanilamide	DMF	water	35	293.15–323.15	https://doi.org/10.1016/j.molliq.2020.114342
sulfamethizole	DMF	water	24	298.15–313.15	https://doi.org/10.3390/ma14205915
Theobromine	DMF	water	12	298.15	https://doi.org/10.3390/pharmaceutics13081118
Theophylline	DMF	water	12	298.15	https://doi.org/10.3390/ijms22147347
Phenacetin	DMF	water	24	298.15–313.15	https://doi.org/10.3390/molecules26134078
nicotinamide	DMF	water	24	298.15–313.15	https://doi.org/10.3390/ijms22147365
Caffeine	DMF	water	48	298.15–313.15	https://doi.org/10.3390/ma15072472
benzamide	DMF	water	24	298.15–313.15	https://doi.org/10.3390/molecules27103323
salicylamide	DMF	water	24	298.15–313.15	https://doi.org/10.3390/molecules27103323
ethenzamide	DMF	water	24	298.15–313.15	https://doi.org/10.3390/molecules27103323
Benzenesulfonamide	DMF	water	24	298.15–313.15	https://doi.org/10.3390/molecules28135008
Paracetamol	DMF	water	24	298.15–313.15	https://doi.org/10.3390/pharmaceutics14122828
Sulfamethoxazole	DMF	water	24	298.15–313.15	https://doi.org/10.3390/molecules29204894
sulfathiazole	DMF	water	24	298.15–313.15	https://apd.umk.pl/diplomas/138379/
sulfamethazine	DMF	water	35	293.15–323.15	https://doi.org/10.3390/molecules29204894
Ferulic acid	DMF	water	11	298.15	https://doi.org/10.3390/molecules30224444
meloxicam	formamide	water	55	293.15–313.15	https://doi.org/10.1016/j.jct.2020.106332
meloxicam	N-methylformamide	water	55	293.15–313.15	https://doi.org/10.1016/j.jct.2020.106332
indomethacin	water	1,4-dioxane	70	293.15–313.15	https://doi.org/10.1016/j.fluid.2010.09.027
sulfadiazine	water	1,4-dioxane	89	293.15–313.15	https://doi.org/10.1016/j.fluid.2015.03.046
aprepitant	water	1,4-dioxane	99	283.15–323.15	https://doi.org/10.1016/j.jct.2020.106170
5,7-Dibromo-8-hydroxyquinoline	water	1,4-dioxane	110	288.15–333.15	https://doi.org/10.1016/j.jct.2020.106138
ribavirin	water	1,4-dioxane	88	283.15–318.15	https://doi.org/10.1016/j.jct.2017.07.027
paclobutrazol	1,4-dioxane	water	121	288.15–313.15	https://doi.org/10.1016/j.jct.2017.05.004
clotrimazole	1,4-dioxane	water	88	278.15–313.15	https://doi.org/10.1016/j.jct.2020.106255
methocarbamol	water	1,4-dioxane	11	298.15	https://doi.org/10.1016/j.molliq.2013.10.012
Salicylic Acid	1,4-dioxane	water	11	298.15	https://pubs.acs.org/doi/10.1021/je800475d
meloxicam	1,4-dioxane	water	80	293.15–313.15	https://pubs.acs.org/doi/10.1021/ie503101h
ketoconazole	1,4-dioxane	water	55	293.20–313.20	https://doi.org/10.1016/j.molliq.2020.112830
Thiamphenicol	1,4-dioxane	water	77	288.15–318.15	https://pubs.acs.org/doi/10.1021/acs.jced.8b00179
Sulfanilamide	1,4-dioxane	water	51	293.15–323.15	https://doi.org/10.1080/00319104.2021.1888382
Sulfapyridine	1,4-dioxane	water	17	298.15	https://raccefyn.co/index.php/raccefyn/article/view/44/32
Sulfisomidine	1,4-dioxane	water	21	298.15	https://raccefyn.co/index.php/raccefyn/article/view/44/32
Sulfamethoxypyridazine	1,4-dioxane	water	18	298.15	https://raccefyn.co/index.php/raccefyn/article/view/44/32
Sulfamethizole	1,4-dioxane	water	43	298.15–313.15	https://doi.org/10.3390/ma14205915
Sulfamethoxazole	1,4-dioxane	water	15	298.15	https://raccefyn.co/index.php/raccefyn/article/view/44/32
Phenacetin	1,4-dioxane	water	89	293.15–313.15	https://doi.org/10.3390/molecules26134078

Solute	Solvent 1	Solvent 2	N	Temperature range [K]	Source
Paracetamol	1,4-dioxane	water	60	293.00–313.00	https://doi.org/10.1021/js980149x
Acetanilide	1,4-dioxane	water	55	293.00–313.00	https://doi.org/10.1021/js980149x
Nalidixic Acid	1,4-dioxane	water	72	283.00–313.00	https://doi.org/10.1021/js980149x
naringenin	1,4-dioxane	water	11	298.15	https://doi.org/10.1007/s10953-016-0526-2
Theobromine	1,4-dioxane	water	12	298.15	https://doi.org/10.3390/pharmaceutics13081118
Theophylline	1,4-dioxane	water	12	298.15	https://doi.org/10.3390/ijms22147347
nicotinamide	1,4-dioxane	water	24	298.15–313.15	https://doi.org/10.3390/ijms22147365
Caffeine	1,4-dioxane	water	48	298.15–313.15	https://doi.org/10.3390/ma15072472
sulfamethazine	1,4-dioxane	water	35	293.15–323.15	https://doi.org/10.3390/molecules29204894
dapsone	1,4-dioxane	water	88	288.15–323.15	https://doi.org/10.1016/j.jct.2018.03.010
triclocarban	1,4-dioxane	water	105	293.15–313.15	https://doi.org/10.1016/j.molliq.2018.09.026
caffeic acid	1,4-dioxane	water	11	298.15	https://doi.org/10.3390/molecules30224444
Ferulic acid	1,4-dioxane	water	11	298.15	https://doi.org/10.3390/molecules30224444
4-Nitrobenzamide	water	DMSO	88	293.15–328.15	https://doi.org/10.1016/j.jct.2019.05.007
d-Histidine	water	DMSO	99	293.15–333.15	https://pubs.acs.org/doi/10.1021/acs.jced.9b01051
adenosine	DMSO	water	77	293.15–323.15	https://doi.org/10.1016/j.jct.2017.07.023
naftopidil	water	DMSO	88	293.15–328.15	https://doi.org/10.1016/j.jct.2019.02.016
gimeracil	water	DMSO	88	293.15–328.15	https://doi.org/10.1016/j.jct.2019.01.026
sinapic acid	water	DMSO	55	298.15–318.15	https://doi.org/10.1016/j.molliq.2016.11.009
2-Aminobenzoic acid	DMSO	Water	30	288.18–308.15	https://doi.org/10.1016/j.molliq.2020.112566
maraviroc	DMSO	Water	77	293.15–323.15	https://doi.org/10.1016/j.jct.2019.106044
Micoflavin	DMSO	Water	66	293.15–318.15	https://pubs.acs.org/doi/10.1021/acs.jced.9b01139
Baricitinib	DMSO	water	55	298.20–323.20	https://doi.org/10.3390/molecules25092124
2-Methoxy-4-nitroaniline	DMSO	water	77	293.15–323.15	https://pubs.acs.org/doi/10.1021/acs.jced.0c00041
pyridazinone	DMSO	water	55	298.20–318.20	https://doi.org/10.3390/molecules25010171
sulfanilamide	DMSO	water	35	293.15–323.15	https://doi.org/10.1016/j.molliq.2020.114342
sulfamethizole	DMSO	water	24	298.15–313.15	https://doi.org/10.3390/ma14205915
Theobromine	DMSO	water	12	298.15	https://doi.org/10.3390/pharmaceutics13081118
Theophylline	DMSO	water	12	298.15	https://doi.org/10.3390/ijms22147347
Phenacetin	DMSO	water	24	298.15–313.15	https://doi.org/10.3390/molecules26134078
nicotinamide	DMSO	water	24	298.15–313.15	https://doi.org/10.3390/ijms22147365
Caffeine	DMSO	water	48	298.15–313.15	https://doi.org/10.3390/ma15072472
benzamide	DMSO	water	24	298.15–313.15	https://doi.org/10.3390/molecules27103323
salicylamide	DMSO	water	24	298.15–313.15	https://doi.org/10.3390/molecules27103323
ethenzamide	DMSO	water	24	298.15–313.15	https://doi.org/10.3390/molecules27103323
Benzenesulfonamide	DMSO	water	24	298.15–313.15	https://doi.org/10.3390/molecules28135008
Paracetamol	DMSO	water	24	298.15–313.15	https://doi.org/10.3390/pharmaceutics14122828
sulfamethoxazole	DMSO	water	24	298.15–313.15	https://doi.org/10.3390/molecules29204894
sulfathiazole	DMSO	water	24	298.15–313.15	https://apd.umk.pl/diplomas/138379/
sulfamethazine	DMSO	water	35	293.15–323.15	https://doi.org/10.3390/molecules29204894
Isotretinoin	DMSO	water	55	298.15–318.15	https://doi.org/10.3390/molecules28207110
caffeic acid	DMSO	Water	11	298.15	https://doi.org/10.3390/molecules30224444
aprepitant	water	acetone	99	283.15–323.15	https://doi.org/10.1016/j.jct.2020.106170
rutaecarpine	water	acetone	99	283.15–323.15	https://doi.org/10.1016/j.jct.2020.106253
Benzoic Acid	acetone	water	63	288.15–318.15	https://pubs.acs.org/doi/10.1021/acs.jced.8b00025
Isomaltulose	water	acetone	55	283.15–323.15	https://pubs.acs.org/doi/10.1021/acs.jced.8b00823
4-Nitrophthalimide	water	acetone	110	278.15–323.15	https://pubs.acs.org/doi/10.1021/acs.jced.0c00479
l-Fucose	acetone	water	55	288.15–308.15	https://pubs.acs.org/doi/10.1021/acs.jced.8b00361
acipimox	water	acetone	56	283.15–318.15	https://doi.org/10.1016/j.molliq.2018.04.009
3-Methyl-6-nitroindazole	acetone	water	88	278.15–328.15	https://pubs.acs.org/doi/10.1021/acs.jced.8b01256
Genistin	acetone	water	64	278.20–313.20	https://pubs.acs.org/doi/10.1021/acs.iecr.5b03393
1-methyl-4-nitropyrazole	water	acetone	81	283.15–323.15	https://doi.org/10.1016/j.molliq.2019.111211
gliclazide	acetone	water	99	278.15–318.15	https://doi.org/10.1016/j.molliq.2020.113258; https://doi.org/10.1016/j.molliq.2020.113425
Theobromine	acetone	water	12	298.15	https://doi.org/10.3390/pharmaceutics13081118
dapsone	acetone	water	110	278.15–323.15	https://doi.org/10.1016/j.molliq.2019.02.023
coumarin	acetone	water	77	273.15–303.15	https://doi.org/10.1080/00319104.2018.1437917
salicin	water	acetone	55	283.15–323.15	https://pubs.acs.org/doi/10.1021/acs.jced.0c00332
Caffeine	acetone	water	48	298.15–313.15	https://doi.org/10.3390/ma15072472
sulfamethazine	water	acetonitrile	189	278.15–318.15	https://doi.org/10.1016/j.fluid.2019.112361
Gallic acid	water	acetonitrile	66	293.15–318.15	https://doi.org/10.1016/j.molliq.2016.07.063
3,5-dibromo-4-hydroxybenzaldehyde	water	acetonitrile	110	278.15–323.15	https://doi.org/10.1016/j.jct.2020.106252
griseofulvin	water	acetonitrile	110	278.15–323.15	https://doi.org/10.1016/j.jct.2020.106250
4-nitropyrazole	water	acetonitrile	187	278.15–318.15	https://doi.org/10.1016/j.jct.2016.08.023
ribavirin	water	acetonitrile	99	278.15–318.15	https://doi.org/10.1016/j.jct.2017.07.027
o-phenylenediamine	acetonitrile	water	165	283.15–318.15	https://doi.org/10.1016/j.jct.2016.10.018
clotrimazole	acetonitrile	water	110	278.15–323.15	https://doi.org/10.1016/j.jct.2020.106255
buprofezin	acetonitrile	water	110	273.15–318.15	https://doi.org/10.1016/j.jct.2019.06.019
acipimox	water	acetonitrile	56	283.15–318.15	https://doi.org/10.1016/j.molliq.2018.04.009
ethylparaben	water	acetonitrile	108	278.15–318.15	https://doi.org/10.1016/j.molliq.2019.110894
ketoconazole	acetonitrile	water	55	293.20–313.20	https://doi.org/10.1080/00319104.2019.1706178
sulfamerazine	acetonitrile	water	189	278.15–318.15	https://doi.org/10.1016/j.molliq.2019.111507
2-Aminobenzoic acid	acetonitrile	water	30	288.18–308.15	https://doi.org/10.1016/j.molliq.2020.112566
Doxofylline	acetonitrile	water	70	278.15–323.15	https://doi.org/10.1016/j.molliq.2020.112952
Paracetamol	acetonitrile	water	55	293.20–313.20	https://doi.org/10.1016/j.molliq.2020.114708
sulfadiazine	acetonitrile	water	55	293.15–313.15	https://doi.org/10.1080/00319104.2019.1594227
lamotrigine	acetonitrile	water	55	293.15–313.15	https://doi.org/10.1080/00319104.2019.1636380
naringenin	acetonitrile	water	11	298.15	https://doi.org/10.1007/s10953-016-0526-2
sulfanilamide	acetonitrile	water	35	293.15–323.15	https://doi.org/10.1016/j.molliq.2020.114342
sulfamethizole	acetonitrile	water	24	298.15–313.15	https://doi.org/10.3390/ma14205915
Phenacetin	acetonitrile	water	24	298.15–313.15	https://doi.org/10.3390/molecules26134078
nicotinamide	acetonitrile	water	24	298.15–313.15	https://doi.org/10.3390/ijms22147365
Caffeine	acetonitrile	water	48	298.15–313.15	https://doi.org/10.3390/ma15072472
sulfamethazine	acetonitrile	water	35	293.15–323.15	https://doi.org/10.1016/j.fluid.2019.112361
chrysin	THF	Water	11	298.15	https://doi.org/10.1016/j.molliq.2016.05.019
gliclazide	THF	water	99	278.15–318.15	https://doi.org/10.1016/j.molliq.2020.113258; https://doi.org/10.1016/j.molliq.2020.113425

Table S2.2. Characteristics of the PhAAc dataset used for model development. N denotes the number of solubility records taken from the cited source, and temperature range refers to the experimental temperature range covered by the corresponding solute–solvent system.

Solute	Solvent 1	Solvent 2	N	Temperature range [K]	Source
caffeic acid	1,4-dioxane	Water	11	298.15	https://doi.org/10.3390/molecules30224444
caffeic acid	DMSO	Water	11	298.15	https://doi.org/10.3390/molecules30224444
caffeic acid	4FM	Water	11	298.15	https://doi.org/10.3390/molecules30224444
Ferulic acid	1,4-dioxane	Water	11	298.15	https://doi.org/10.3390/molecules30224444
Ferulic acid	DMF	Water	11	298.15	https://doi.org/10.3390/molecules30224444
Ferulic acid	4-formylmorpholine	Water	11	298.15	https://doi.org/10.3390/molecules30224444
rosmarinic acid	water	Methyl Acetate	35	293.15–313.15	https://pubs.acs.org/doi/10.1021/acs.jced.6b00008
rosmarinic acid	water	ethanol	42	293.15–318.15	https://doi.org/10.1016/j.molliq.2016.01.061
rosmarinic acid	water	methanol	42	293.15–318.15	https://doi.org/10.1016/j.molliq.2016.01.061
rosmarinic acid	water	Ethyl Acetate	35	293.15–313.15	https://pubs.acs.org/doi/10.1021/acs.jced.6b00008
Syringic acid	water	Methanol	99	283.15–323.15	https://pubs.acs.org/doi/10.1021/acs.jced.7b00333
Gallic acid	water	propan-1-ol	66	293.15–318.15	https://doi.org/10.1016/j.molliq.2016.07.063
Gallic acid	water	propan-2-ol	66	293.15–318.15	https://doi.org/10.1016/j.molliq.2016.07.063
Gallic acid	water	ethanol	72	293.15–318.15	https://doi.org/10.1016/j.jct.2012.06.022
Gallic acid	water	methanol	36	293.15–318.15	https://doi.org/10.1016/j.molliq.2013.07.015
Gallic acid	water	acetonitrile	66	293.15–318.15	https://doi.org/10.1016/j.molliq.2016.07.063
Vanillic acid	Water	methanol	66	293.15–318.15	https://doi.org/10.1016/j.molliq.2016.04.095
trans-cinnamic acid	ethanol	water	81	288.15–328.15	https://doi.org/10.1016/j.molliq.2018.09.131
trans-cinnamic acid	methanol	water	81	288.15–328.15	https://doi.org/10.1016/j.molliq.2018.09.131
Ferulic acid	isopropanol	water	55	298.20–318.20	https://doi.org/10.1111/jphp.12786
sinapic acid	Carbitol	water	55	298.15–318.15	https://doi.org/10.1007/s10973-020-09451-y
sinapic acid	water	DMSO	55	298.15–318.15	https://doi.org/10.1016/j.molliq.2016.11.009
Salicylic Acid	Ethanol	water	17	298.15	https://pubs.acs.org/doi/10.1021/je800475d
Salicylic Acid	Methanol	water	7	298.15	https://pubs.acs.org/doi/10.1021/je800475d
Salicylic Acid	Ethanol	Ethyl Acetate	11	298.15	https://pubs.acs.org/doi/10.1021/je800475d
Salicylic Acid	1,4-Dioxane	water	11	298.15	https://pubs.acs.org/doi/10.1021/je800475d
Vanillic Acid	water	Ethanol	77	293.15–323.15	https://pubs.acs.org/doi/10.1021/acs.jced.5b00619
sinapic acid	ethylene glycol	water	55	298.15–318.15	https://doi.org/10.1016/j.molliq.2021.118057
Benzoic Acid	Ethanol	hexane	81	288.15–328.15	https://pubs.acs.org/doi/10.1021/acs.jced.8b00025
Benzoic Acid	isopropyl alcohol	hexane	80	288.15–323.15	https://pubs.acs.org/doi/10.1021/acs.jced.8b00025
Benzoic Acid	chloroform	hexane	80	288.15–323.15	https://pubs.acs.org/doi/10.1021/acs.jced.8b00025
Benzoic Acid	acetone	hexane	70	288.15–318.15	https://pubs.acs.org/doi/10.1021/acs.jced.8b00025
Benzoic Acid	acetone	water	63	288.15–318.15	https://pubs.acs.org/doi/10.1021/acs.jced.8b00025
Benzoic acid	chloroform	diethyl ether	11	298.15	https://pubs.acs.org/doi/10.1021/ja02263a014
5-aminosalicylic acid	N-methyl-2-pyrrolidone	ethanol	55	293.20–313.20	https://doi.org/10.1016/j.molliq.2020.112774
mesalazine	1-propanol	water	55	293.20–313.20	https://doi.org/10.1016/j.molliq.2019.112436
mesalazine	carbitol	ethanol	55	293.20–313.20	https://doi.org/10.1016/j.molliq.2020.114763
mesalazine	ethylene glycol	water	55	293.20–313.20	https://doi.org/10.1016/j.molliq.2020.114597
mesalazine	propylene glycol	ethanol	40	293.20–313.20	https://doi.org/10.1016/j.molliq.2020.112714
mesalazine	N-methyl-2-pyrrolidone	water	55	293.20–313.20	https://doi.org/10.1016/j.molliq.2020.113143
mesalazine	2-propanol	water	55	293.20–313.20	https://doi.org/10.1016/j.molliq.2020.112474
dl-malic acid	water	ethanol	88	298.15–333.15	https://doi.org/10.1016/j.fluid.2014.06.017
3-methyl-4-nitrobenzoic acid	1,4-dioxane	methanol	165	283.15–318.15	https://doi.org/10.1016/j.jct.2016.10.019
3-methyl-4-nitrobenzoic acid	NMP	methanol	165	283.15–318.15	https://doi.org/10.1016/j.jct.2016.10.019
3-methyl-4-nitrobenzoic acid	DMF	methanol	165	283.15–318.15	https://doi.org/10.1016/j.jct.2016.10.019
hydroxyacetic acid	Ethanol	Ethyl acetate	81	273.15–313.15	https://doi.org/10.1016/j.jct.2017.01.004
2-Aminobenzoic acid	DMF	Water	30	288.18–308.15	https://doi.org/10.1016/j.molliq.2020.112566
2-Aminobenzoic acid	DMSO	Water	30	288.18–308.15	https://doi.org/10.1016/j.molliq.2020.112566
2-Aminobenzoic acid	Acetonitrile	Water	30	288.18–308.15	https://doi.org/10.1016/j.molliq.2020.112566
succinic acid	ethanol	Water	96	278.15–333.15	https://doi.org/10.1016/j.molliq.2016.12.042
malonic acid	2-propanol	ethyl acetate	64	278.15–313.15	https://doi.org/10.1007/s10953-019-00853-7
ketoprofen	propylene glycol	water	55	293.15–313.15	https://doi.org/10.1016/j.fluid.2010.03.031
Ibuprofen	Ethanol	Propylene glycol	30	293.15–313.15	https://doi.org/10.1016/j.fluid.2007.07.076
Ibuprofen	Propylene glycol	water	30	293.15–313.15	https://doi.org/10.1007/s10953-007-9228-0
Ibuprofen	Ethanol	water	38	293.15–313.15	http://www.latamjpharm.org/trabajos/26/3/LAJOP_26_3_1_4_6YFYLM039U.pdf
Ketoprofen	methanol	water	11	298.15	https://doi.org/10.1080/00319104.2016.1140763
Ibuprofen	methanol	water	11	298.15	https://doi.org/10.1080/00319104.2016.1140763
d-Histidine	water	DMF	99	293.15–333.15	https://pubs.acs.org/doi/10.1021/acs.jced.9b01051
d-Histidine	water	DMSO	99	293.15–333.15	https://pubs.acs.org/doi/10.1021/acs.jced.9b01051
d-Histidine	water	NMP	99	293.15–333.15	https://pubs.acs.org/doi/10.1021/acs.jced.9b01051
d-Histidine	water	ethanol	99	293.15–333.15	https://pubs.acs.org/doi/10.1021/acs.jced.9b01051
Zaltoprofen	1,4-Dioxane	Ethanol	48	288.15–323.15	https://pubs.acs.org/doi/10.1021/acs.jced.9b01180
Zaltoprofen	2-methoxyethanol	ethanol	60	278.15–323.15	https://pubs.acs.org/doi/10.1021/acs.jced.9b01180
Zaltoprofen	2-methoxyethanol	Ethyl Acetate	60	278.15–323.15	https://pubs.acs.org/doi/10.1021/acs.jced.9b01180
Zaltoprofen	2-Ethoxyethanol	Ethyl Acetate	60	278.15–323.15	https://pubs.acs.org/doi/10.1021/acs.jced.9b01180
Artesunate	water	methanol	99	278.15–318.15	https://pubs.acs.org/doi/10.1021/acs.jced.8b00988
Artesunate	water	ethanol	99	278.15–318.15	https://pubs.acs.org/doi/10.1021/acs.jced.8b00988
Artesunate	water	isopropanol	99	278.15–318.15	https://pubs.acs.org/doi/10.1021/acs.jced.8b00988
Artesunate	water	propylene glycol	99	278.15–318.15	https://pubs.acs.org/doi/10.1021/acs.jced.8b00988
Naproxen	Ethanol	Propylene glycol	30	293.15–313.15	https://doi.org/10.1016/j.fluid.2007.07.076
Naproxen	Ethanol	water	55	293.15–313.15	https://doi.org/10.1080/00319100701313862
Naproxen	Propylene glycol	water	30	293.15–313.15	https://doi.org/10.1007/s10953-007-9228-0
Adipic Acid	Cyclohexanol	Cyclohexanone	36	303.00–353.00	https://pubs.acs.org/doi/10.1021/acs.jced.5b00880
indomethacin	propylene glycol	water	70	293.15–313.15	https://doi.org/10.1016/j.fluid.2011.11.001
indomethacin	ethanol	propylene glycol	44	293.15–308.15	https://doi.org/10.1016/j.molliq.2013.02.008
Terephthalaldehydic acid	NMP	water	99	283.15–323.15	https://pubs.acs.org/doi/10.1021/acs.jced.8b01262
Terephthalaldehydic acid	Methanol	water	99	283.15–323.15	https://pubs.acs.org/doi/10.1021/acs.jced.8b01262
Terephthalaldehydic acid	ethanol	water	99	283.15–323.15	https://pubs.acs.org/doi/10.1021/acs.jced.8b01262
Terephthalaldehydic acid	Isopropanol	water	99	283.15–323.15	https://pubs.acs.org/doi/10.1021/acs.jced.8b01262
Stearic acid	ethanol	ethyl acetate	77	293.15–323.15	https://doi.org/10.1016/j.molliq.2019.112101
2,5-Furandicarboxylic Acid	water	acetic acid	118	303.55–363.15	https://pubs.acs.org/doi/10.1021/acs.jced.7b00927
para-Methoxyphenylacetic Acid	Propanol-2	Toluene	90	283.15–323.15	https://pubs.acs.org/doi/10.1021/acs.jced.8b00271
4-aminobutyric acid	methanol	water	99	283.15–323.15	https://doi.org/10.1016/j.jct.2016.07.014
maleic acid	ethyl acetate	acetone	60	285.15–324.25	https://doi.org/10.1016/j.tca.2012.03.023
Zaltoprofen	1,4-Dioxane	Ethyl Acetate	48	288.15–323.15	https://pubs.acs.org/doi/10.1021/acs.jced.9b01180
1,4-butanedioic acid	1-propanol	water	25	293.20–333.20	https://doi.org/10.1016/j.fluid.2012.04.019
Ascorbic Acid	water	1-propanol	12	298.15–308.15	https://pubs.acs.org/doi/10.1021/je900687y
Adipic Acid	Acetic Acid	water	44	303.20–333.20	https://pubs.acs.org/doi/10.1021/je301202v
gamma-aminobutyric acid	ethanol	water	36	288.20–313.20	https://pubs.acs.org/doi/10.1021/ie901517b

Solute	Solvent 1	Solvent 2	N	Temperature range [K]	Source
Nalidixic Acid	1,4-Dioxane	water	72	283.00–313.00	https://doi.org/10.1021/js980149x
o-toluic acid	Ethanol	water	30	293.15–313.15	https://jbiochemtech.com/article/thermodynamic-solvation-parameters-for-saturated-benzoic-acid-and-some-of-its-derivatives-in-binary-mixtures-of-ethanol-and-water
p-toluic acid	Ethanol	water	30	293.15–313.15	https://jbiochemtech.com/article/thermodynamic-solvation-parameters-for-saturated-benzoic-acid-and-some-of-its-derivatives-in-binary-mixtures-of-ethanol-and-water
2-chlorobenzoic acid	Ethanol	water	30	293.15–313.15	https://jbiochemtech.com/article/thermodynamic-solvation-parameters-for-saturated-benzoic-acid-and-some-of-its-derivatives-in-binary-mixtures-of-ethanol-and-water
4-chlorobenzoic acid	Ethanol	water	30	293.15–313.15	https://jbiochemtech.com/article/thermodynamic-solvation-parameters-for-saturated-benzoic-acid-and-some-of-its-derivatives-in-binary-mixtures-of-ethanol-and-water
malonic acid	acetone	acetonitrile	64	278.15–313.15	https://doi.org/10.1002/ceat.201700227
indomethacin	water	1,4-dioxane	70	293.15–313.15	https://doi.org/10.1016/j.fluid.2010.09.027
vitamin C	Ethanol	isopropanol	44	293.15–323.15	https://doi.org/10.1016/j.jct.2018.02.005
vitamin C	Methanol	isopropanol	44	293.15–323.15	https://doi.org/10.1016/j.jct.2018.02.005
vitamin C	Methanol	ethanol	44	293.15–323.15	https://doi.org/10.1016/j.jct.2018.02.005
vitamin C	Water	isopropanol	44	293.15–323.15	https://doi.org/10.1016/j.jct.2018.02.005
vitamin C	Water	Ethanol	44	293.15–323.15	https://doi.org/10.1016/j.jct.2018.02.005
Naproxen	ethyl acetate	ethanol	55	293.15–313.15	https://doi.org/10.1016/j.fluid.2012.02.009
indomethacin	ethanol	water	55	293.15–313.15	https://doi.org/10.1016/j.fluid.2011.06.016
Naproxen	methanol	water	11	298.15	https://doi.org/10.1080/00319104.2016.1140763
Indomethacin	Ethanol	acetone	11	298.15	https://pubs.acs.org/doi/10.1021/acs.jced.8b00536
Indomethacin	Ethanol	acetonitrile	11	298.15	https://pubs.acs.org/doi/10.1021/acs.jced.8b00536
vanillic acid	4-formylmorpholine	water	11	298.15	This work

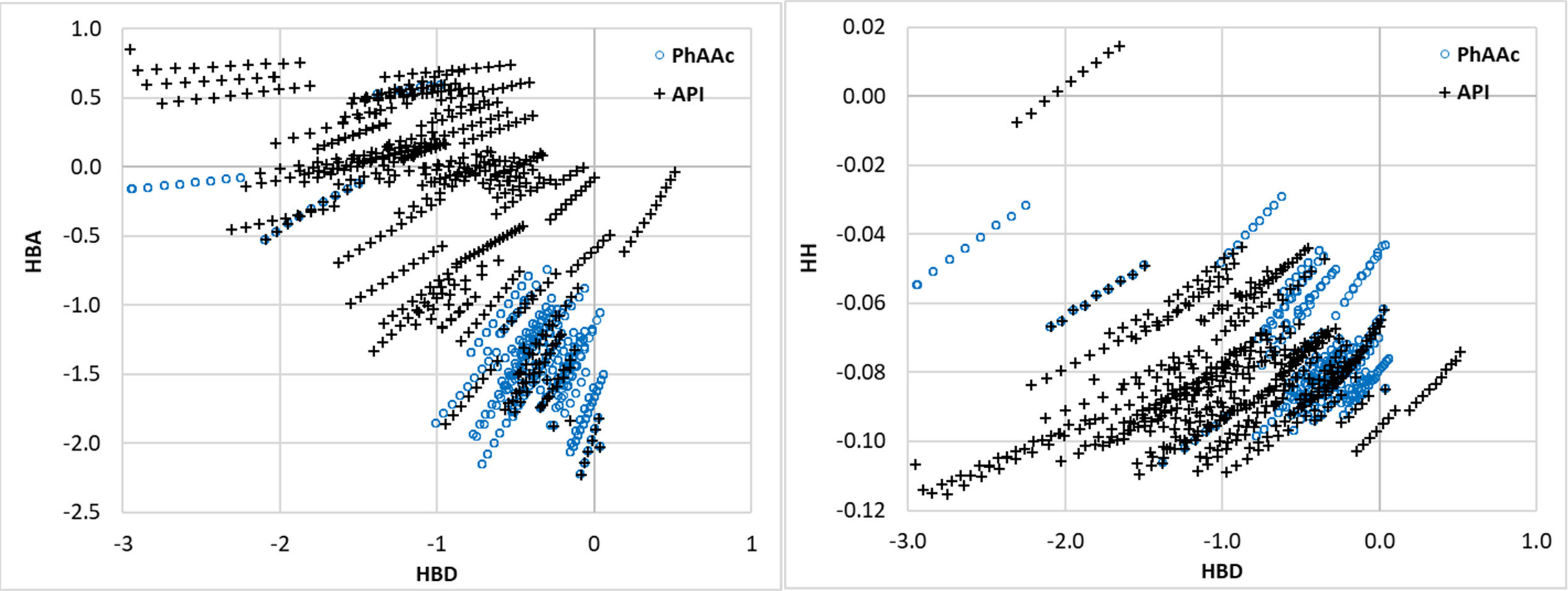

Figure S2.1. Dataset diversity illustrated by the relationship between hydrogen-bond donor character (HBD) and (a) hydrogen-bond acceptor character (HBA) or (b) hydrophobic character (HH) for the API and PhAAc datasets. HBD = HBD1 + HBD2 + HBD3 + HBD4, HBA = HBA1 + HBA2 + HBA3 + HBA4, and HH = HH1 + HH2 + HH3 + HH4.

S3. Descriptors used for ML

Table S3.1. Detailed explanations of the descriptors used in the study. The acronyms are consistent with the terminology used in the spreadsheet in the Supplementary Materials.

Descriptor	Acronym	Explanation
$\log(x^{est})$	RefSol	COSMO-RS derived solubility collected as decadal logarithm of mole fraction using reference solvent approach
μ_{API}^{sat}	mu_sat	chemical potential of API under saturated conditions
E_{API}^{tot}	solute_Etot	total API interaction energy under saturated conditions
E_{API}^{misfit}	solute_Emisfit	electrostatic (misfit) contribution to API energy under saturated conditions
E_{API}^{HB}	solute_Ehbond	hydrogen-bonding contribution to API intermolecular interaction energies under saturated conditions
E_{API}^{VdW}	solute_Evdw	non-bonding contribution to API intermolecular interaction energies under saturated conditions
E_{SOLV}^{tot}	solv_Etot	total interaction energies of SOLV $E_{solv}^{tot} = \sum_{i=1}^{N=3(4)} x_i^* \cdot E_i^{tot} \cdot$
E_{SOLV}^{Misfit}	solv_Emisfit	electrostatic interaction energies of SOLV $E_{SOLV}^{Misfit} = \sum_{i=1}^{N=3(4)} x_i^* \cdot E_i^{Misfit} \cdot$
E_{SOLV}^{HB}	solv_Ehbond	hydrogen bonding interaction energies of SOLV $E_{SOLV}^{HB} = \sum_{i=1}^{N=3(4)} x_i^* \cdot E_i^{HB} \cdot$
E_{SOLV}^{vdW}	solv_Evdw	non-bonding interaction energies of SOLV $E_{SOLV}^{vdW} = \sum_{i=1}^{N=3(4)} x_i^* \cdot E_i^{vdW} \cdot$
μ_{SOLV}^{sat}	solv_mu	chemical potential of SOLV under saturated conditions $\mu_{SOLV}^{sat} = \sum_{i=1}^{N=3(4)} x_i^* \cdot \mu_i \cdot$
$d\mu^{sat}$	rel_mu	relative value of chemical potential (μ): $d\mu^{sat} = \mu_{API}^{sat} - \mu_{SOLV}^{sat}$
dE_{SOLV}^{tot}	rel_Etot	relative values of total interaction energy in the saturated system $dE_{SOLV}^{tot} = E_{API}^{tot} - E_{SOLV}^{tot}$
dE_{SOLV}^{Misfit}	rel_Emisfit	relative value of the electrostatic contribution to intermolecular interaction energies: $dE_{SOLV}^{Misfit} = E_{API}^{Misfit} - E_{SOLV}^{Misfit}$
dE_{SOLV}^{HB}	rel_Ehbond	relative value of the hydrogen bonding contribution to intermolecular interaction energies $dE_{SOLV}^{HB} = E_{API}^{HB} - E_{SOLV}^{HB}$
dE_{SOLV}^{vdW}	rel_Evdw	relative value of the non-bonding contribution to intermolecular interaction energies: $dE_{SOLV}^{vdW} = E_{API}^{vdW} - E_{SOLV}^{vdW}$

Table S3.2. Detailed explanations of the σ-potential related descriptors used in the study. The acronyms are consistent with the terminology used in the spreadsheet in the Supplementary Materials.

Descriptor	Acronym	Explanation
$\sigma(HBD1)_{API}$	API-HBD1	$HBD1_{API}$ (from –0.03e/Å^2 to -0.025 e/Å^2)
$\sigma(HBD2)_{API}$	API-HBD2	$HBD2_{API}$ (from –0.025e/Å^2 to –0.020 e/Å^2)
$\sigma(HBD3)_{API}$	API-HBD3	$HBD3_{API}$ (from –0.020e/Å^2 to –0.015 e/Å^2)
$\sigma(HBD4)_{API}$	API-HBD4	$HBD4_{API}$ (from –0.015e/Å^2 to –0.010 e/Å^2)
$\sigma(HH1)_{API}$	API-HH1	$HH1_{API}$ (from –0.010e/Å^2 to –0.005 e/Å^2)
$\sigma(HH2)_{API}$	API-HH2	$HH2_{API}$ (from –0.005e/Å^2 to 0.000 e/Å^2)
$\sigma(HH3)_{API}$	API-HH3	$HH3_{API}$ (from 0.000e/Å^2 to +0.005 e/Å^2)
$\sigma(HH4)_{API}$	API-HH4	$HH4_{API}$ (from +0.005e/Å^2 to +0.010 e/Å^2)
$\sigma(HBA1)_{API}$	API-HBA1	$HBA1_{API}$ (from +0.010e/Å^2 to +0.015 e/Å^2)
$\sigma(HBA2)_{API}$	API-HBA2	$HBA2_{API}$ (from +0.015e/Å^2 to +0.020 e/Å^2)
$\sigma(HBA3)_{API}$	API-HBA3	$HBA3_{API}$ (from +0.020e/Å^2 to +0.025 e/Å^2)
$\sigma(HBA4)_{API}$	API-HBA4	$HBA4_{API}$ (from +0.025e/Å^2 to +0.030 e/Å^2)
$\sigma(HBD1)_{SOLV}$	SOLV-HBD1	$HBD1_{SOLV}$ (from –0.03e/Å2 to –0.025 e/Å2)
$\sigma(HBD2)_{SOLV}$	SOLV-HBD2	$HBD2_{SOLV}$ (from –0.025e/Å2 to –0.020 e/Å2)
$\sigma(HBD3)_{SOLV}$	SOLV-HBD3	$HBD3_{SOLV}$ (from –0.020e/Å2 to –0.015 e/Å2)
$\sigma(HBD4)_{SOLV}$	SOLV-HBD4	$HBD4_{SOLV}$ (from –0.015e/Å2 to –0.010 e/Å2)
$\sigma(HH1)_{SOLV}$	SOLV-HH1	$HH1_{SOLV}$ (from –0.010e/Å2 to –0.005 e/Å2)
$\sigma(HH2)_{SOLV}$	SOLV-HH2	$HH2_{SOLV}$ (from –0.005e/Å2 to 0.000 e/Å2)
$\sigma(HH3)_{SOLV}$	SOLV-HH3	$HH3_{SOLV}$ (from 0.000e/Å2 to +0.005 e/Å2)
$\sigma(HH4)_{SOLV}$	SOLV-HH4	$HH4_{SOLV}$ (from +0.005e/Å2 to +0.010 e/Å2)
$\sigma(HBA1)_{SOLV}$	SOLV-HBA1	$HBA1_{SOLV}$ (from +0.010e/Å2 to +0.015 e/Å2)
$\sigma(HBA2)_{SOLV}$	SOLV-HBA2	$HBA2_{SOLV}$ (from +0.015e/Å2 to +0.020 e/Å2)
$\sigma(HBA3)_{SOLV}$	SOLV-HBA3	$HBA3_{SOLV}$ (from +0.020e/Å2 to +0.025 e/Å2)
$\sigma(HBA4)_{SOLV}$	SOLV-HBA4	$HBA4_{SOLV}$ (from +0.025e/Å2 to +0.030 e/Å2)
$\sigma(HBD1)_{API-SOLV}$	d-HBD1	ΔHBD1 (from –0.03e/Å^2 to –0.025 e/Å^2)
$\sigma(HBD2)_{API-SOLV}$	d-HBD2	ΔHBD2 (from –0.025e/Å2 to –0.020 e/Å2)

Descriptor	Acronym	Explanation
$\sigma(HBD3)_{API-SOLV}$	d-HBD3	ΔHBD3 (from –0.020e/Å2 to –0.015 e/Å2)
$\sigma(HBD4)_{API-SOLV}$	d-HBD4	ΔHBD4 (from –0.015e/Å2 to –0.010 e/Å2)
$\sigma(HH1)_{API-SOLV}$	d-HH1	ΔHH1 (from –0.010e/Å2 to –0.005 e/Å2)
$\sigma(HH2)_{API-SOLV}$	d-HH2	ΔHH2 (from –0.005e/Å2 to 0.000 e/Å2)
$\sigma(HH3)_{API-SOLV}$	d-HH3	ΔHH3 (from 0.000e/Å2 to +0.005 e/Å2)
$\sigma(HH4)_{API-SOLV}$	d-HH4	ΔHH4 (from +0.005e/Å2 to +0.010 e/Å2)
$\sigma(HBA1)_{API-SOLV}$	d-HBA1	ΔHBA1 (from +0.010e/Å2 to +0.015 e/Å2)
$\sigma(HBA2)_{API-SOLV}$	d-HBA2	ΔHBA2 (from +0.015e/Å2 to +0.020 e/Å2)
$\sigma(HBA3)_{API-SOLV}$	d-HBA3	ΔHBA3 (from +0.020e/Å2 to +0.025 e/Å2)
$\sigma(HBA4)_{API-SOLV}$	d-HBA4	ΔHBA4 (from +0.025e/Å2 to +0.030 e/Å2)

S4. Feature Selection Across SGKF Folds

Table S4.1. Fold-wise feature selection results across SGKF folds for the PhAAc dataset (XGBoost).

fold	best_outer_mae	n_features	best_features	model_params	R^2	RMSD	MAE
0	0.199276	19	['RefSol', 'dE_HB_sat', 'dE_vdW_sat', 'E1_tot_sat', 'E1_HB_sat', 'E1_vdW_sat', 'E_Misfit_solvent', 'E_HB_solvent', 'E_vdW_solvent', 'd_HBD1', 'd_HBD2', 'd_HBD4', 'd_HH1', 'd_HH2', 'd_HH3', 'd_HBA1', 'd_HBA2', 'd_HBA3', 'd_HBA4']	{'n_estimators': 320, 'learning_rate': 0.023927528765580644, 'max_depth': 7, 'subsample': 0.69971689165955, 'colsample_bytree': 0.8549719605992826, 'reg_lambda': 0.029204338471814112}	0.870	0.301	0.199
1	0.249179	5	['RefSol', 'E1_vdW_sat', 'd_HH2', 'd_HH4', 'd_HBA4']	{'n_estimators': 696, 'learning_rate': 0.025841935310839222, 'max_depth': 4, 'subsample': 0.82272050498334, 'colsample_bytree': 0.9744619096643123, 'reg_lambda': 0.6083019192425827}	0.931	0.351	0.249
2	0.235735	4	['RefSol', 'E_tot_solvent', 'd_HH2', 'd_HH3']	{'n_estimators': 525, 'learning_rate': 0.016666983286066417, 'max_depth': 3, 'subsample': 0.9795542149013333, 'colsample_bytree': 0.9862528132298237, 'reg_lambda': 1.7123375973163988}	0.848	0.324	0.236
3	0.234679	17	['RefSol', 'dE_vdW_sat', 'E1_HB_sat', 'E1_vdW_sat', 'E_tot_solvent', 'E_HB_solvent', 'E_vdW_solvent', 'd_HBD1', 'd_HBD2', 'd_HH1', 'd_HH2', 'd_HH3', 'd_HH4', 'd_HBA1', 'd_HBA2', 'd_HBA3', 'd_HBA4']	{'n_estimators': 221, 'learning_rate': 0.016666983286066417, 'max_depth': 5, 'subsample': 0.9795542149013333, 'colsample_bytree': 0.9718790609370292, 'reg_lambda': 1.7123375973163988}	0.929	0.306	0.235
4	0.18716	7	['RefSol', 'E_tot_solvent', 'E_HB_solvent', 'd_HH2', 'd_HH3', 'd_HBA2', 'd_HBA4']	{'n_estimators': 683, 'learning_rate': 0.011674531719265543, 'max_depth': 4, 'subsample': 0.9795542149013333, 'colsample_bytree': 0.9862528132298237, 'reg_lambda': 1.7123375973163988}	0.934	0.258	0.187

Table S4.2. Fold-wise feature selection results across SGKF folds for the API dataset (LightGBM)

fold	best_outer_mae	n_features	best_features	model_params	r2	rmse	mae
0	0.33173	8	['RefSol', 'dE_Misfit_sat', 'dE_HB_sat', 'E1_tot_sat', 'E1_vdW_sat', 'd_HBD1', 'd_HH1', 'API_HBA4']	{'n_estimators': 736, 'learning_rate': 0.020496381951116718, 'num_leaves': 15, 'subsample': 0.8963074471016818, 'colsample_bytree': 0.9942601816442402}	0.907	0.441	0.332
1	0.397571	10	['RefSol', 'dE_Misfit_sat', 'dE_HB_sat', 'dE_vdW_sat', 'E1_vdW_sat', 'E_vdW_solvent', 'd_HBD1', 'd_HH1', 'API_HH2', 'API_HBA4']	{'n_estimators': 114, 'learning_rate': 0.07489770465258357, 'num_leaves': 22, 'subsample': 0.6849356442713105, 'colsample_bytree': 0.8912865394447438}	0.857	0.525	0.398
2	0.363162	6	['RefSol', 'dE_HB_sat', 'dE_vdW_sat', 'E1_vdW_sat', 'd_HBD1', 'd_HH1']	{'n_estimators': 185, 'learning_rate': 0.04407984038169244, 'num_leaves': 19, 'subsample': 0.9637281608315128, 'colsample_bytree': 0.9652962210225885}	0.817	0.542	0.363
3	0.336062	7	['RefSol', 'dE_HB_sat', 'dE_vdW_sat', 'E1_vdW_sat', 'd_HBD1', 'd_HH1', 'API_HBA2']	{'n_estimators': 344, 'learning_rate': 0.06729596646792112, 'num_leaves': 18, 'subsample': 0.8034282764658811, 'colsample_bytree': 0.9630265895704372}	0.801	0.572	0.336
4	0.40284	6	['RefSol', 'dE_vdW_sat', 'E1_vdW_sat', 'd_HBD1', 'd_HH1', 'API_HBA4']	{'n_estimators': 392, 'learning_rate': 0.019452208847287395, 'num_leaves': 20, 'subsample': 0.7350460685614512, 'colsample_bytree': 0.9771638815650077}	0.802	0.598	0.403
